# Small Vessels Are a Big Problem in Neurodegeneration and Neuroprotection

**DOI:** 10.3389/fneur.2019.00889

**Published:** 2019-08-16

**Authors:** Şefik Evren Erdener, Turgay Dalkara

**Affiliations:** ^1^Institute of Neurological Sciences and Psychiatry, Hacettepe University, Ankara, Turkey; ^2^Department of Neurology, Faculty of Medicine, Hacettepe University, Ankara, Turkey

**Keywords:** capillary, microcirculation, pericytes, oxygen extraction, flow heterogeneity, neuroprotection, neurodegeneration

## Abstract

The cerebral microcirculation holds a critical position to match the high metabolic demand by neuronal activity. Functionally, microcirculation is virtually inseparable from other nervous system cells under both physiological and pathological conditions. For successful bench-to-bedside translation of neuroprotection research, the role of microcirculation in acute and chronic neurodegenerative disorders appears to be under-recognized, which may have contributed to clinical trial failures with some neuroprotectants. Increasing data over the last decade suggest that microcirculatory impairments such as endothelial or pericyte dysfunction, morphological irregularities in capillaries or frequent dynamic stalls in blood cell flux resulting in excessive heterogeneity in capillary transit may significantly compromise tissue oxygen availability. We now know that ischemia-induced persistent abnormalities in capillary flow negatively impact restoration of reperfusion after recanalization of occluded cerebral arteries. Similarly, microcirculatory impairments can accompany or even precede neural loss in animal models of several neurodegenerative disorders including Alzheimer's disease. Macrovessels are relatively easy to evaluate with radiological or experimental imaging methods but they cannot faithfully reflect the downstream microcirculatory disturbances, which may be quite heterogeneous across the tissue at microscopic scale and/or happen fast and transiently. The complexity and size of the elements of microcirculation, therefore, require utilization of cutting-edge imaging techniques with high spatiotemporal resolution as well as multidisciplinary team effort to disclose microvascular-neurodegenerative connection and to test treatment approaches to advance the field. Developments in two photon microscopy, ultrafast ultrasound, and optical coherence tomography provide valuable experimental tools to reveal those microscopic events with high resolution. Here, we review the up-to-date advances in understanding of the primary microcirculatory abnormalities that can result in neurodegenerative processes and the combined neurovascular protection approaches that can prevent acute as well as chronic neurodegeneration.

## Introduction

Cerebral microcirculation is a fundamental element for proper cerebral functioning since it is the main transport and distribution system for oxygen and nutrients that fuel the high and continuously changing metabolic demand of the brain tissue. The anatomical and physiological features of small vessels, which mainly consist of vasculature <100 μm in diameter (1), where most of the metabolic exchange takes place, are considerably different from large vessels and these differences make them difficult to study experimentally and virtually inaccessible in human beings. Over the last years, advances in imaging technologies and modeling made such investigations possible and increased the recognition of microcirculatory pathologies in acute and chronic neurodegenerative conditions, revealing novel mechanisms and potential therapeutic strategies. In this review, the structural and functional principles of cerebral microcirculation and its pathophysiological relevance will be discussed.

## Capillary Dysfunction

### The Complexity of Microcirculation

**Figure 1:**
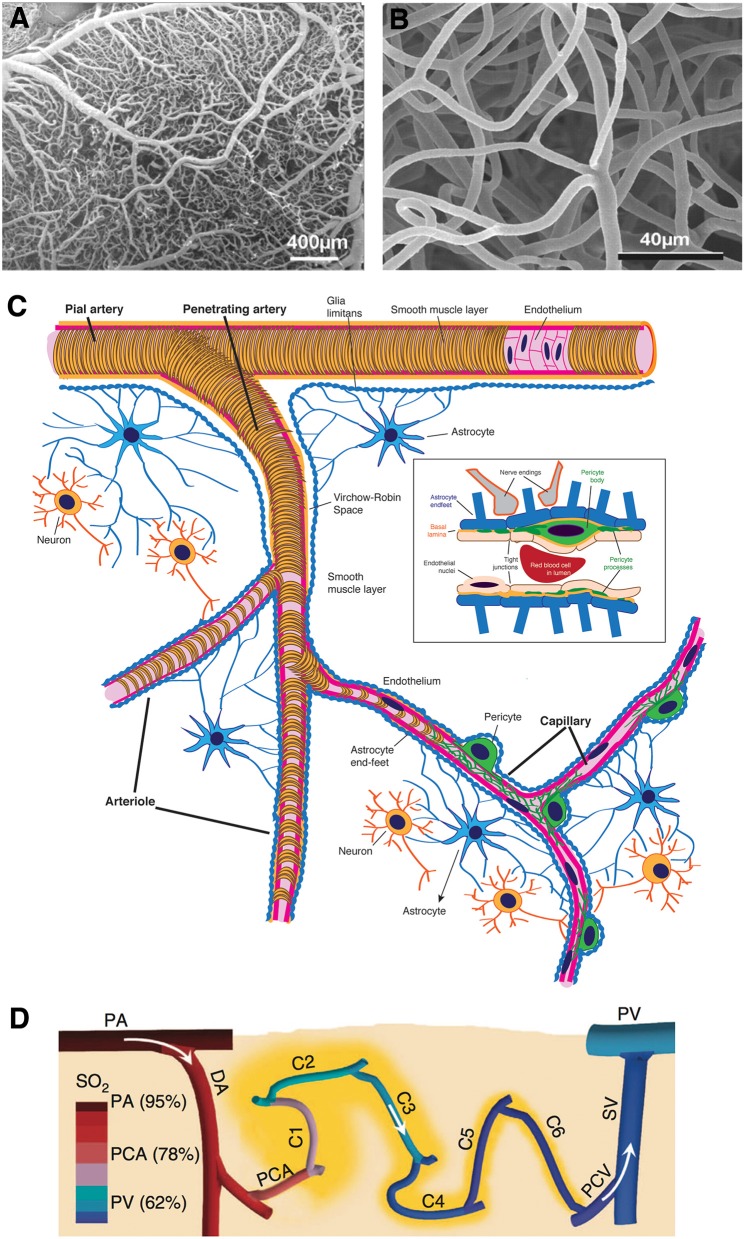
Morphological features of cerebral microcirculation. **(A,B)** Dense external coverage of the surface by pial arteries and veins which are interconnected via anastomoses and collateral vessels then gives rise to a complex meshwork of capillaries, composed of segments about 60–70 μm in length. **(C)** Penetrating arteries originating from pial vasculature dive into the cortical tissue without forming any further anastomoses. Immediately surrounded by the cerebrospinal fluid-filled spaces called *perivascular spaces* or *Virchow-Robin spaces* along their course, penetrating arteries then give rise an extensive tree of small vessels as they branch into arterioles (30–100 μm diameter), precapillary arterioles (10–30 μm), and capillaries (<10 μm), respectively. As arterioles turn into capillaries, the perivascular space disappears, making the capillary wall adjacent to the parenchyma. **(D)** Capillaries branch out for 5–6 times on average (2) as they release oxygen into the tissue, until they converge on postcapillary venules that drain into ascending veins. (**A,B** reproduced by permission from: PNAS, Meyer et al. (3) ^©^2008 National Academy of Sciences. **(D)** reprinted by permission from: Nature Communications, Sakadžić et al. (2) ^©^2014 Springer Nature).

The brain is a vascular organ as much as it is neuronal. The anatomy of cerebral vasculature is unique and optimized to provide continuous blood supply to the brain. Extensive anastomoses between pial vessels on the surface ensure that extra blood can be shuffled to the activated brain area demanding more energy. As illustrated in Figure 1, progressive branching of penetrating arteries into a high-density meshwork of capillaries allows for adequate delivery of nutrients, such that there is a capillary within 10–20 μm of every neuron (4, 5). The density of capillary mesh correlates with neuronal density and differs between cortical layers or brain regions. Unlike simplified illustrations, capillary networks have a complex structure, branching in various directions, forming loops, and curls to optimally match the tissue demand.

Tissue blood flow is provided by the pressure gradient across the microcirculatory bed vascular resistance, which is largely determined by the vascular diameter and blood viscosity (6). The focal brain activity is highly variable; hence, regional metabolic requirements change fast and continuously. This requires a precise spatial and temporal control of the local blood flow. Consequently, flow changes are tightly coupled to neuronal activity through a set of mechanisms integrated within the *neurovascular unit* (7, 8). This *neurovascular coupling* can be mediated by both feed-forward (neuronal activity itself directly regulates blood flow) or feed-back (the metabolic consequences of increased activity regulates blood flow) mechanisms (8, 9).

Studying microcirculation is challenging in animals as well as humans because of the structural and functional complexity of the system. This has caused a delay in understanding role of microcirculatory dysfunction in neurological disorders, which is more relevant than previously thought. The microcirculatory dynamics are fast and heterogeneous, so we need tools that can acquire data with high spatial (in microns) and temporal (in milliseconds) resolution. Most of the available information on capillary flow and oxygenation is based on magnetic resonance imaging (MRI) as well as direct *in vivo* microscopic imaging that can be performed only in animals. Two-photon microscopy (TPM) through a cranial window in rodents provides high resolution angiogram of the capillary network and, red blood cell (RBC) flux and speed estimation within individual capillaries (12–14). Phosphorescence lifetime microscopy (PLIM) adds assessment of the oxygen with subcapillary resolution both in microvasculature and cerebral tissue (2, 15–22). Optical coherence tomography (OCT), which is sensitive to motion of scattering particles enable visualizing RBC flow label-free. Unlike TPM that images a limited area, OCT-angiography allows visualizing hundreds of capillaries simultaneously through the cortical mantle (10, 23–26). In MRI, a voxel of 1 mm^3^ reflects merely an average of many capillaries but MRI has the advantage of imaging whole brain non-invasively (27).

Studies with these instruments have revealed that the increase in capillary blood flow occurs slightly before or in synchrony with upstream vessels, suggesting a direct role to the microcirculation to coordinate functional hyperemia response (29–32). Under resting state, most of the oxygen release into the tissue occurs at the precapillary arteriole level, while distal capillaries become an additional a site of oxygen extraction during functional hyperemia (2). Especially during baseline conditions, the capillary pO_2_ distribution is highly heterogeneous, some capillaries have very low oxygen, while not necessarily making the tissue around them critically hypoxic but particularly vulnerable to fluctuations in blood flow and/or increase in metabolic demand (2, 16).

### Microcirculation Plays an Active Role in Neurovascular Coupling

**Figure 2:**
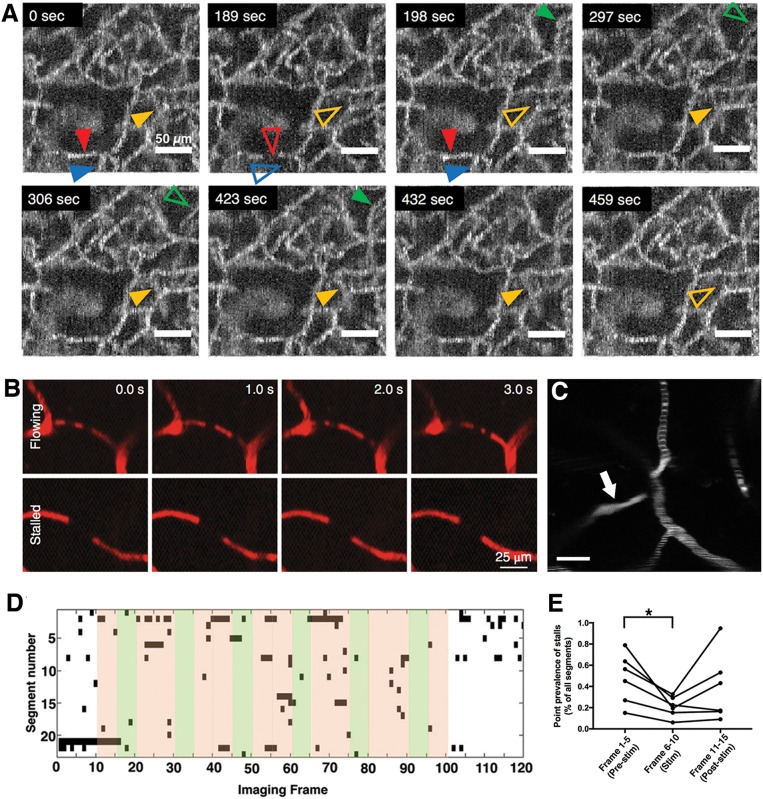
Irregularity and heterogeneity in capillary flow **(A)** OCT angiogram time-series identify capillary segments with stalling red blood cells. Individual segments (arrowheads) with temporary interruptions of RBC flux simply lose OCT angiogram signal. Hollow arrowheads indicate a stalled capillary segment (10). **(B)** Two photon microscopy time series with fluorescent labeled plasma can identify flowing and stalled capillaries based on the motion of unlabeled cells as seen in black (11). **(C)** A single time-point two-photon angiogram of a set of capillary branches at ~100 μm below cortical surface shows heterogeneous distribution of RBC flux. Segments with higher flow have thinner and denser RBC-bands whereas slower flow is indicated by thicker and more scarce bands. One segment (arrow) has no RBCs flowing but is still filled with fluorescent labeled plasma. Scalebar: 20 μm) **(D)** Matrix plot of individual stall events in a region of interest, acquired during a functional stimulation experiment, with a frame period of 9 s. Every black points denotes a stall in a particular capillary. Green shades indicate whisker stimulation. **(E)** The frequency of capillary stalls is dynamically modulated during functional stimulation; stall prevalence was significantly lower during functional hyperemia. ^*^Statistical significance (*p* < 0.05). (**A,D,E** reproduced by permission from: JCBFM, Erdener et al. (10); **(B)** reprinted by permission from: Nature Neuroscience, Cruz Hernandez et al. (11), ^©^2019 Springer Nature).

Models for blood flow simulation and oxygen transport / extraction (34–38) suggest that capillary flow patterns are important to determine tissue oxygen availability (37–39). While cerebral capillaries are normally almost always perfused with plasma during rest and activated conditions, the flow speeds of RBCs, like spatial oxygen distribution, are heterogeneous at baseline. This is caused by variability in capillary diameters, pressure gradients, and non-Newtonian fluid characteristics of blood, resulting in different RBC and plasma fractions in different capillaries that can affect the flow patterns (40) (Figure 2C). In a recent study, monitoring of capillaries frame-by-frame by OCT angiography revealed frequent temporary stalls that lasted for few seconds-minutes in individual capillaries (10) (Figure 2A). These temporal flow fluctuations can be caused by RBCs or white blood cells (WBCs) getting stuck or slowed down inside capillaries (10, 11, 41) because of their relatively large size compared to the capillary lumen (Figure 2B). They are more prevalent in capillaries closer to the distal end of the microcirculation, where pressure gradients are lower and RBCs are slower, and also where endothelial adhesion molecules like intercellular cell adhesion molecule-1 (ICAM-1) have higher expression (42). During functional activation, RBC flow speeds increase to make flow distribution in capillary bed more homogeneous and the dynamic stalls diminish (10) (Figures 2D,E). This homogeneous transformation in oxygenation and flow speeds is essential for optimum oxygen extraction. Because, in case of heterogeneous flows, some capillaries have higher flow velocity compared to the neighboring vessels and they act as thoroughfare channels, shunting highly-oxygenated RBCs through the microcirculation to leave little time for oxygen release (37). This is important because it has been shown that increasing overall arterial blood flow to a capillary bed with heterogeneous flow distribution can paradoxically worsen oxygen availability to the tissue (38, 43, 44). For this reason, functional blood flow responses in the brain need to be actively regulated at the capillary level and not solely by dilating arterioles. This function is realized by structural and metabolic collaboration between neurons, endothelia, astrocytes and pericytes, the building blocks of the neurovascular unit.

Endothelium is the common inner lining of all types of vasculature, including capillaries. In the central nervous system (CNS) tight junctions between endothelial cells form the critical layer of the blood brain barrier (BBB) that controls material passage into the parenchyma. In the healthy setting, endothelium prevents paracellular plasma leakage by tight junctions and there is a low level of transcytotic vesicle trafficking through endothelial cells (45, 46). Oxygen and carbon dioxide, on the other hand, can freely diffuse across the capillary wall bidirectionally (46–48), allowing rapid gas exchange. The endothelium, basal membrane, and astrocyte end feet together form the capillary wall and the BBB (49). Degradation of BBB either due to basal lamina changes, endothelial or astrocyte damage is a common feature of many neurodegenerative disorders which have microcirculatory pathologies. While there is no smooth muscle layer in the capillary wall, there is another contractile cell, the pericyte located at the periphery of microvessel wall and embedded into the basal membrane layers (50). Pericytes have a contractile apparatus, which is similar to that of smooth muscle cells. Pericyte processes concentrically surround the capillaries, enabling them to regulate the capillary diameter (51, 52). Pericytes have also been shown to contribute to BBB formation and maintenance, to immunological modulation, angiogenesis, and capillary reorganization (53–55). Pericytes therefore are attractive candidates for capillary-level control of neurovascular coupling. Indeed, pericyte relaxation during functional activation has been shown to precede or being closely coincident with functional hyperemia (30, 51). The strong interactions between pericytes and endothelial cells and astrocytes further underline their strategic role in microcirculation (56, 57).

Astrocyte end-feet around microvessels are critical elements for neuron-microvessel communication. Nearby neuronal activity is sensed by astrocyte processes as extracellular adenosine triphosphate (ATP) and glutamate increase around synapses. Calcium signals triggered in astrocytes lead to cyclooxygenase activation and release of vasodilatory prostaglandins from end-feet surrounding the capillaries (58, 59). This is one of the basic mechanisms for translation of neuronal activity to vasoactive regulation. Other mechanisms involving metabolic and paracrine interactions within elements of the tightly integrated neurovascular unit are also present and contribution of each varies between brain regions. For example, astrocyte processes are also metabolically coupled to excitatory synapses; they rapidly breakdown glycogen to release lactate as an energy supply to neurons (60). An intriguing feature of the astrocyte network is the gap junctions between end-feet integrated to the capillary wall. Activity-induced calcium signaling can rapidly propagate around microvasculature to provide a coordinated response in a given area (59, 61–63). Capillary pericytes readily respond to the local release of vasodilatory prostaglandins (30). Endothelial cells, which are electrically coupled to other endothelial cells and to smooth muscle cells via gap junctions (64–67), have also been shown to propagate membrane depolarizations and calcium waves along the vasculature, releasing nitric oxide (NO) in consequence (68), adding to the coordinated microvascular regulation. Interneurons are also directly involved in vasoactive regulation. For example, stimulation of GABAergic interneurons has been shown to produce a biphasic blood flow response, hyperemia, followed by vasoconstriction, similar to the somatosensory activation response, by releasing NO and neuropeptide Y, respectively (69–72). Interestingly, red blood cells within the capillary lumen themselves can also take active role in capillary flow regulation by releasing ATP in response to focal acidosis, hypoxia, shear stress, or mechanical stimuli (73–76). The ATP release can be initiated by opening of pannexin channels or voltage-dependent anion channels in erythrocytes (75, 77). ATP, by binding to purinergic receptors can trigger NO or prostaglandin release from the endothelium (73) generating a local vasodilatory response to match the oxygen demand in the stagnated microenvironment (73).

The mechanisms of neurovascular regulation should be recognized in the setting of an integrated vascular network, where different levels of microcirculation may work in concert in addition to a tight compartmentalization. For example, arterioles appear to favor the vasoactive signals coming from interneurons coding the activity in a larger cohort of neurons in contrast to capillary regulation responding to very focal demand translated by adjacent astrocytes (69, 78). Arterioles also respond to signals coming up through the endothelia to accommodate the blood volume demand by the downstream microcirculatory bed (58, 59, 61–63).

### Microcirculatory Failure

Since oxygen availability in the tissue relies on the distribution of capillary flow patterns and their heterogeneity, the absolute quantitative level of cerebral blood flow (CBF) may not always reflect accurate supply-demand match at microscopic level. In routine clinical practice of cerebrovascular medicine, main focus is on the evaluation of adequate arterial flow and luminal patency, as they can be easily imaged by widely available radiological techniques. In the setting of a CBF drop, particularly to ischemic levels (below ~20 ml/100 g/min in humans) (79), there is a prominent decrease in tissue oxygen tension paralleled by a relative increase in oxygen extraction. However, experimental and computer simulation data indicate that the tissue oxygen availability may be compromised even in the absence of a flow-limiting condition (37, 38). This arises from “capillary dysfunction,” causing excessive flow heterogeneity in the capillary bed and failing to homogenize in response to increasing functional demand. This heterogeneity causes certain capillary segments to transit oxygenated RBCs too fast for enough oxygen to be released to tissue (36, 44). A classic example of this phenomenon is the hyperperfusion syndrome (38, 80, 81), a temporary excessive hyperemia after recanalization of an occluded cerebral artery, which paradoxically results in low tissue oxygenation (38). Very slow flux rates in some capillaries may also lead to a reduced oxygen delivery due to insufficient number of RBCs transiting. Any factor introducing irregularity to the capillary flow, results in a higher standard deviation in distribution of RBC velocities and/or transit times across the microvascular bed, i.e., the capillary transit time heterogeneity (CTH). However, it should be noted that increased CTH may passively increase with high mean transit time (MTT), the average time that contrast bolus traverse between arterial and venous ends (43). Importantly, the relative transit time heterogeneity (CTH/MTT), i.e., the coefficient of variation of transit times, can distinguish between true microcirculatory heterogeneity and a passive increase in CTH with low CBF and elevated MTT. The relationship between CBF, CTH, oxygen extraction fraction (OEF), and tissue oxygen can be evaluated with computational models (36, 82, 83). These models show that, CTH is, counter intuitively; homogenized with very low flow rates to maximize O_2_ extraction in poorly perfused tissue (37, 82). The detailed evaluation of mathematical models is beyond the scope of this review and the reader is referred to excellent papers on this subject (84, 85). MRI data can be used to prepare MTT, CTH, and OEF maps with use of appropriate mathematical models (27). These tools have been useful for evaluation of capillary dysfunction in acute ischemic stroke and Alzheimer's disease patients (36, 38, 43, 82, 86).

The capillary dysfunction can arise from almost any element within the neurovascular unit. A thin (0.5 μm) glycoprotein coating in the luminal side of the endothelium, called glycocalyx, facilitates passage of blood cells (87–89). Glycocalyx can degrade very fast with excessive inflammatory stimuli like lipopolysaccharides, hyperglycemia, ischemia, and oxidative stress (90). A degraded glycocalyx exposes underlying endothelium to physical interaction with blood cells. This usually results in a higher degree of cellular adhesion. Each cellular plug, whether permanent or transient, can then increase resistance in that particular segment, change pressure gradient, flux, and hematocrit distribution in nearby capillary bed and cause irregular hemodynamics. A relative increase in hematocrit in other capillaries within the same network may increase the tendency for plugging.

Endothelial cell dysfunction can significantly contribute to dysregulated capillary flow. This dysfunction includes a reduction in endothelium-derived nitric oxide availability. Moreover, gap-junction uncoupling between endothelial cells increases functional shunting by introducing irregularities to the microvascular cross-sectional profiles, caused by desynchronized regulation of smooth muscle or pericyte tone (91, 92). A dysfunctional endothelium is also more prone to leukocyte adhesion, as nitric oxide is a well-recognized modulator of leukocyte adhesion (92–94). Leukocyte plugs and resultant flow stalls in capillaries can unfavorably affect flow patterns and, the release of leukocyte-derived oxygen radicals or cytokines can damage the endothelium and overlying glycocalyx. BBB breakdown may accompany a damaged and dysfunctional endothelium. Endothelium, besides these barrier-forming and material exchange functions, is also involved in immunological regulation, leukocyte adhesion and regulation of thrombosis as well as vascular tone (95).

Pericytes maintain the BBB, interact with immune cells (96) and, since they are contractile, regulate capillary flow distribution within the microvascular network. Both constriction or dilation of capillaries mediated by pericytes can result in stalls in cellular flow or functional shunts (32). Pericytes contract or constrict with changes in oxygen tension, with exposure to reactive oxygen radicals, noradrenaline, ATP and endothelin (28, 30, 53, 97), while glutamate, adenosine, lactate, nitric oxide, and C-type natriuretic peptide cause relaxation (30, 97–99). Besides active contractile changes, loss of pericytes themselves can cause capillary dysfunction (100). Amyloid-beta has been shown to cause pericyte degeneration (101, 102), which may be a possible link between neurodegenerative disorders and microcirculatory dysfunction. Hyperglycemia directly causes pericyte death or migration, hence, leaky newly-formed microvessels are typical features of diabetic retinopathy (99, 103). Pericytes have been shown to migrate along or away from vasculature following traumatic injury (104, 105). With pathological stimuli, pericyte process coverage over capillary wall can decrease, further disrupting microvascular physiology (106, 107).

Finally, the physicochemical properties of plasma or blood cells themselves can introduce microcirculatory heterogeneity. As noted above, RBCs and WBCs have large diameters compared to capillaries therefore they need to squeeze through the narrow capillary lumen, significantly making passage challenging and vulnerable to disturbances (108). Even subtle capillary constrictions can therefore dramatically affect RBC perfusion distribution in a capillary network (36, 82). Changes in cell count, size, stiffness, flexibility, and adhesion can affect the capillary transit (8). In experimental models of polycythemia vera and essential thrombocythemia, it has been shown that individual capillaries are clogged by RBCs, and then platelets, affecting the CBF (41). Rheological properties, like deformability of RBCs can affect microcirculatory efficiency as seen in diabetes (109). Blockade of capillaries by activated neutrophils are also observed in cerebral ischemia (110–113). Dehydration and elevated homocysteine can increase plasma viscosity and/or hematocrit, causing formation of RBC aggregates, and capillary plugs (38, 114). It should be noted that plasma may continue to flow though even when there is no cellular flow in a capillary, unfavorably affecting tissue survival as plasma provides glucose to the hypo-oxygenized tissue, promoting lactate production and tissue acidosis (28, 115–117).

The tissue can tolerate a certain degree of capillary dysfunction by regulating upstream arteriolar flow (either increasing or decreasing) to optimize oxygen extraction (37, 44, 118–120). A reduction in resting CBF and suppression of functional hemodynamic responses can be a part of this compensation (37, 121). But after a certain degree of CTH, usually accompanied by a higher level of capillary diameter irregularities, glycocalyx damage, elevated blood viscosity, leukocyte adhesion, and excessive red blood cell stalls, the supplied oxygen cannot sustain neuronal homeostasis any more (36). At this level of severe capillary dysfunction, where CBF is close to the ischemic threshold, slight changes in metabolic needs, systemic blood pressure, leukocyte count, or blood viscosity may cause appearance of hypoxic/ischemic neurological symptoms (36, 37). These events can also cause irreversible morphological changes in microcirculation, like capillary pruning, or appearance of string capillaries with no functional lumen (122–124). It should be noted that all these pathological events described above could occur in the absence of a flow-limiting condition, like arterial occlusion, or stenosis.

## Microcirculatory Dysfunction in Neurodegeneration

### Small Vessel Disease

Small vessel disease is a clinical and imaging phenomenon caused by different etiologies and characterized by pathological changes in vasculature with a diameter <100 μm, including, arterioles, venules, and capillaries (1, 125). It is a common cause of cognitive impairment, gait problems and disability in the elderly (125). Uncontrolled hypertension or diabetes are among the leading risk factors for this chronic vasculopathy characterized with concentric smooth muscle thickening, especially in penetrating vessels as well as pericyte degeneration, basal membrane thickening, endothelial, and astrocyte end-feet swelling in capillaries (38, 126–129). Pro-inflammatory conditions in the endothelium increases tendency to leukocyte adhesion and activation in this pathologic setting (130–134). The slowly progressive worsening of microcirculatory structure and function may result in neuronal loss, brain atrophy, and the white matter changes detected as leukoaraiosis or diffuse white matter hyperintensities with MRI (135, 136). While capillary-level damage may insidiously progress over time, acute vascular events, like spontaneous rupture, or thrombosis of already damaged arteriolar branches (137–141), leading to stepwise clinical deterioration (1). It is not surprising that either the chronic progressive vasculopathy or acute ischemic events superimposed on this is a primary cause of microcirculatory dysfunction, the details of which are outlined in the next section.

### Ischemic Stroke

**Figure 3:**
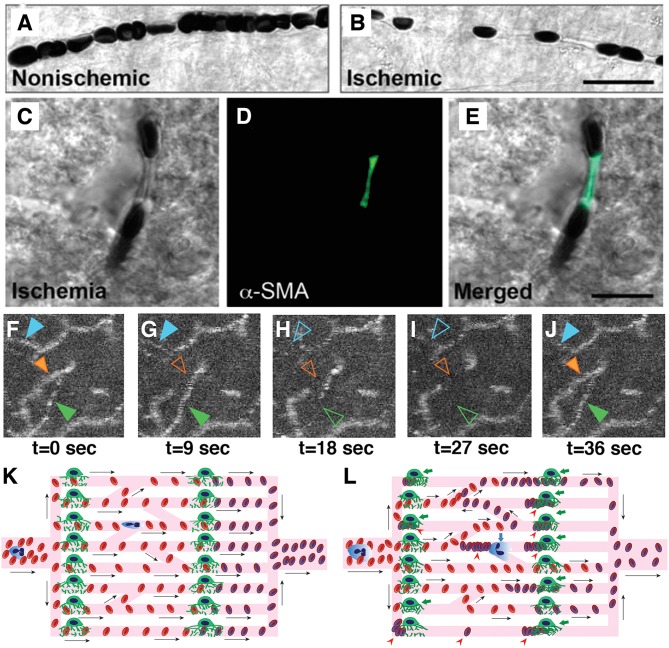
Capillary dysfunction in ischemic stroke and flow-limiting conditions. **(A,B)** Differential interference contrast (DIC) microscopy images illustrate frequent interruptions in the erythrocyte column in an ischemic capillary contrary to a continuous row of erythrocytes flowing through a non-ischemic capillary. Scale bar: 20 μm. **(C–E)** The constricted segments colocalized with α-smooth muscle actin (α-SMA) immunoreactive pericytes. Scale bar: 10 μm. **(F–J)** Very high frequency of dynamic RBC flow stalls in ischemic penumbra shown with OCT angiogram time-series (Manuscript^*^ in preparation). **(K)** Ideally, capillary flow should be homogeneous across a capillary bed to optimize oxygen extraction. Arrows indicate direction of cell motion. **(L)** Pericyte contractions, and increased plugging of leukocytes and red blood cells as a result of ischemia-induced capillary dysfunction, introduce severe heterogeneity into the microcirculation, resulting in redistributions of flow, and pathological shunting. This can profoundly reduce the oxygen delivery into the tissue, even if the total plasma-perfused capillary count and absolute arterial input is the same. Green arrows indicate constricted pericytes, red arrowheads indicate stagnant red blood cells, blue arrow indicates a plugged leukocyte. Deoxygenated RBCs are darker and bluish in color. [**A–E** reproduced by permission from: Nature Medicine, Yemisci et al. (28)]. (^*^Manuscript by authors: Erdener SE, Tang J, Kilic K, Postnov D, Giblin JT, Kura S, Chen A, Vayisoglu T, Sakadzic S, Schaffer CB and Boas DA).

Currently, the only approved treatment of an acute ischemic stroke is prompt recanalization within few hours either by mechanical thrombectomy or thrombolysis with intravenous or intra-arterial tissue plasminogen activator. At the present, this treatment approach only takes large vessel recanalization into account. However, tissue reperfusion is usually incomplete, hence oxygen extraction is not optimal even after complete recanalization (38). In the ischemic brain, pericytes contract in response to hypoxia and reactive oxygen species (28). This luminal narrowing and irregularities can easily block cellular passage, while plasma may continue to flow. Recanalization of the artery does not resolve this microvascular dysfunction as the capillary constrictions are sustained, leading to the no-reflow phenomenon (142, 143) (Figures 3A–E). The quality of capillary perfusion after recanalization is indeed a better indicator of functional outcome than recanalization itself (144, 145). If the capillary flow patterns are extremely dysfunctional and heterogeneous, the recanalization may in fact introduce more functional shunting, paradoxically reducing the oxygen availability (36, 38, 146).

In the ischemic brain, activated neutrophils can also temporarily plug capillaries with their large size and stiff cytoplasm, and can disturb the RBC transit (108). As a clinical evidence, leukocytosis and a high neutrophil fraction are bad prognostic factors in ischemic stroke (147–150). Stroke incidence as well as stroke-related mortality is higher during acute infections (36, 151). The dynamic microcirculatory dysfunction may continue after stroke onset, potentially contributing to infarct expansion over the subsequent days despite recanalization (152–155). One essential way to prevent infarct growth appears to reverse any capillary flow disturbance in addition to large artery recanalization. It is also becoming clear that the definition of ischemic tissue based solely on the level of CBF decrease is insufficient and the capillary flow patterns must also be taken into account (36).

The ischemia-induced microvascular dysfunction progresses in a graded fashion. With a temporary decrease in perfusion pressure in a normal and compliant microvasculature, the cells transiting the microvasculature slow down, since there is less driving force due to lower pressure gradient. Accordingly, MTT values increase. The CTH calculated based on the standard deviation of transit times in multiple pathways across the capillary bed proportionally increases as well. Thereby, the relative transit heterogeneity (RTH = CTH/MTT) remains unchanged and oxygen delivery to the tissue can still be sufficient to match the demand. However, with ongoing ischemia, structural irregularities, like constrictions, and glycocalyx damage in capillaries emerge, making cellular passage complicated (Figures 3F–L). This time, the increase in CTH will be higher than what can be mathematically expected from prolongation of MTTs. The disproportional increase in CTH will yield to higher RTH values, pointing to a specific dysfunction in microcirculation that can result in suboptimal oxygen extraction for the ischemic tissue. The above simplified progressive microcirculatory deterioration is the most likely scenario taking place in the ischemic penumbra, corresponding to a potentially salvageable tissue with timely and efficient recanalization, whereas it can also undergo permanent damage with increasing microvascular damage and oxygenation failure (82). In the most severe form of ischemia, complete capillary occlusions that do not allow even passage of plasma emerge. This end-stage level of capillary dysfunction, paradoxically leads to homogenization of capillary transit times measured from remaining patent ones in the severely ischemic tissue that is destined to infarct regardless of recanalization (82). This low CTH probably reflects flow in maximally dilated flowing capillaries and does not take into account the occluded ones. The phrasing may at first sound at odds with the capillary flow homogenization during normal functional hyperemia that provides optimal oxygen extraction (37). Although it may be a similar neurovascular coupling effort for the tissue to optimize blood flow using the capillaries left available (82), the absolute amount of blood passing through the tissue this time is simply very low, decreasing cerebral blood volume below the viability threshold (156).

Chronic cerebral hypoperfusion, even if it doesn't cause full-blown ischemia, can have long-term effects on microcirculatory structure. Experimental OCT data in mice with carotid artery occlusion show a local micro-heterogeneity of capillary flow and oxygen supply even at non-ischemic global blood flow levels (49). The functional changes are accompanied by histological signs of microglia and astrocyte activation along with capillary dilatation, remodeling, increased tortuosity, and even amyloid-beta accumulation (157–159). These structural pathologies can further contribute to the progression of capillary dysfunction. Recently, severity of carotid stenosis have been associated with accumulation of cortical cerebral microinfarcts and poor cognitive performance, even in patients who did not experience acute ischemic stroke (160). Preoperative capillary transit time heterogeneity measured by MRI, an indicator for capillary dysfunction, predicts the functional independency (i.e., Modified Rankin Scale) following endovascular treatment in patients with symptomatic bilateral high-grade carotid stenosis (161).

### Subarachnoid Hemorrhage

Subarachnoid hemorrhage shares many common pathophysiological features with cerebral ischemia, because the blood products in cerebrospinal fluid (CSF) can trigger arteriolar and microvascular vasospasms (162). Hemoglobin breakdown products and concomitant formation of reactive oxygen species by iron released from hemoglobin (163) as well as release of vasoconstrictive mediators like endothelin-1 and thromboxane into the CSF may play roles in vasospasm (39, 164). Vasospasms emerge usually a few days after the initial hemorrhage. Like in cerebral ischemia, there is profound increase in capillary transit time heterogeneity after subarachnoid hemorrhage, caused by microarteriolar constrictions (165), reactive oxygen radicals, astrocyte end-feet swelling, leukocyte increase, and activation (39). There are numerous capillaries either without RBCs but with plasma, or with stationary RBCs in them (166). Capillaries with smaller diameters have been shown to have less probability to be perfused with RBCs (167). Because of these disturbances in microcirculation, attempts to relax the vasospasm in large arteries are not very effective. The microcirculatory pathology may begin even before gross vasospasm settles in. Within a few minutes after experimental subarachnoid hemorrhage, there is a heterogeneous slowing down of capillary flow with prominent irregularities, even though there is some dilation in penetrating vessels and precapillary arterioles (168, 169). Dilatory response to CO_2_ is lost and an inverse neurovascular coupling response, i.e., constriction instead of expected hyperemia with sensory stimulation is observed 24 h after experimental subarachnoid hemorrhage (170). Whether this flow suppression is an outcome of pathologically high capillary heterogeneity or is a compensatory CBF decrease to optimize oxygen extraction is currently unknown (39).

### Traumatic Brain Injury/Chronic Traumatic Encephalopathy

Ischemic injury is a well-known histological hallmark of traumatic contusion injuries (171). A reduction in global CBF almost always accompanies a contusion or traumatic hematoma. If the injury is severe, vasospasms in large vessels can emerge similar to subarachnoid hemorrhage, causing multifocal infarcts. Elevated intracranial pressure due to traumatic injury in animal models caused severely disturbed capillary flow and limited oxygen and solute extraction due to heterogeneity and shunts in the capillary bed (37, 172). This can be partly explained by a direct negative effect of increased intracranial pressure on cerebral perfusion pressure. The decrease in CBF can last for 12 h and functional blood flow responses are usually suppressed during this period (173, 174). Histologically, there is evidence of astrocyte end-feet swelling, pericyte contraction, and increased expression of smooth muscle actin (174). Also, pericytes have been shown to detach from the vessel wall early after the traumatic insult (105), which might contribute to blood-brain dysfunction in capillary bed (175). Capillaries have been shown to be the major site of vascular leakage in animal models after cortical trauma (176). Increased inflammatory protein expression and activation of leukocytes promote cellular adhesion, therefore limiting RBC passage and causing functional capillary stalls and shunts (108, 174). Leukocytosis, like in ischemic stroke, is associated with poor outcome following traumatic brain injury (172).

Interestingly, a gross contusion, hematoma, or intracranial pressure increase is not necessary for microcirculatory abnormalities to develop. A single mild concussion in mice can also decrease CBF 30–40% up to 24 h, as measured with diffuse correlation spectroscopy (177). With repetitive injuries, the oligemic response can extend to 72 h (177). The microcirculatory disturbances induced by mild injuries are independent of increases in intracranial pressure (178). Instead, cortical spreading depolarizations (CSD) multifocally triggered after such insults correlate with neuronal injury and cerebral microbleeds (172). CSDs are self-propagating waves of intense neuroglial depolarization, followed by prolonged suppression of neural activity. Whereas, CSDs associated with migraine aura are benign, repetitive CSDs can cause tissue injury in the setting of subarachnoid hemorrhage or cause infarct expansion during cerebral ischemia, in the form of peri-infarct spreading depolarizations (179–184). In a healthy brain, CSDs can initiate a hyperemic response followed by a long-term oligemia but, in an already ischemic brain, the hyperemia is reversed to oligemia during the depolarization phase (185). CSD waves cause sustained disturbances in capillary flow, causing massive, and heterogeneous changes in erythrocyte velocities (186). Pericytes contract during CSD in response to an increase in cytoplasmic calcium (187). Neurovascular coupling can disappear for hours, causing a supply-demand mismatch, contributing to tissue injury (186, 187). The reduced (inverse) CBF response to increased metabolic load during massive depolarization could help increase O_2_ extraction by slowing flux rates (186).

Repetitive concussions are associated with chronic traumatic encephalopathy (CTE), a cause of early onset dementia and psychiatric disorders especially in boxers or American football players and also in military veterans exposed to blast injuries (188). Autopsy brain samples from CTE patients and also rodent models have evidence of microcirculatory pathology, characterized with swollen astrocytic end-feet and processes forming tangles around capillaries, blood-brain barrier breakdown, and perivascular deposition of hyperphosphorylated tau especially in deep cortical regions (189–192). Even a single blast exposure or concussive cortical impact in mice has been shown to trigger a progressive tauopathy with evidence of microvascular injury and neurodegeneration, which started in the area of exposure first but spread distally over months (193–195).

### Alzheimer's Disease

A reduction in CBF is observed early in Alzheimer's disease (AD) patients and this has been proposed as a predictor of progression to overt AD from mild cognitive impairment (196–198). Many vascular risk factors like coronary heart disease, dyslipidemia, hypertension are associated with AD (199). The underlying mechanisms and possible impact of the reduction in CBF and microcirculatory abnormalities in the pathogenesis of neurodegeneration are under investigation to better characterize the cause-effect relationship. In mouse models, impaired cerebral perfusion has been shown to stimulate amyloid-beta deposition. Tau protein can also be hyperphosphorylated under hypoxic conditions, even after brief and temporary episodes (200). After global brain ischemia, expression of tau protein increases in CA1 area of hippocampus in mice, one of the most severely affected areas in AD related to memory functions (201). Interestingly, perivascular zones around penetrating arterioles, the bottlenecks of the cerebral blood supply, are one of the initial areas showing tau and amyloid beta deposition (202).

Electron microscopy has identified capillary wall damage, basal membrane thickening and pericyte degeneration in autopsy brains from AD patients (203). In MRI studies, cognitive performance and level of cortical atrophy were found to be associated with low CBF, high CTH, and low oxygen extraction (86, 204). Similar dynamic abnormalities have also been found in transgenic AD mice, with insufficient flow homogenization, lower resting CBF, and reduced cortical oxygen availability (205).

**Figure 4:**
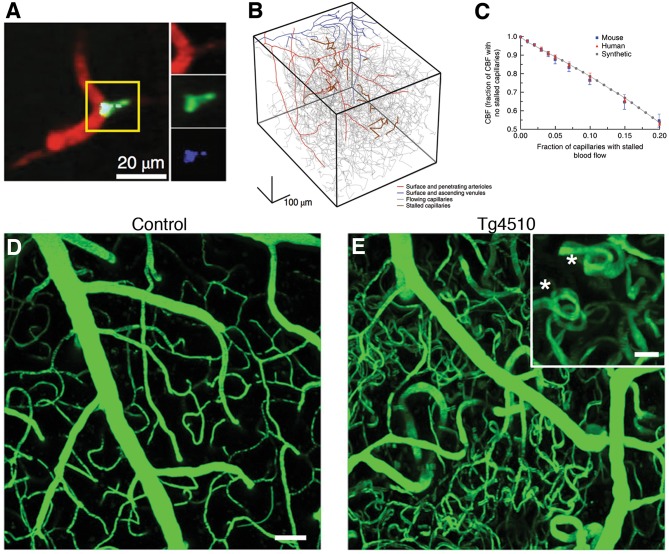
Microcirculatory changes in experimental models of Alzheimer's disease. **(A)** There is increased fraction of stalled capillaries in double-transgenic mice overexpressing amyloid-beta (APP/SW1) due to increased plugging by leukocytes (Green: rhodamine-G labeled leukocyte, red: Texas-red labeled plasma). **(B)** Vascular network tracings show distribution of stalled capillaries. **(C)** Although the fraction of stalled capillaries may seem small, computer simulations on capillary networks show a prominent decrease in overall cerebral blood flow with gradual introduction of stalls. **(D,E)** In another model, 15 month-old mice overexpressing pathological form of hyperphosphorylated tau (Tg4510) show abnormal capillary morphology, number and density (spiral shapes as shown with asterisks(^*^)). Scale bar 50 μm (inset: 20 μm) There is increased number of stagnant of leukocytes also in these capillaries (not shown here). (**A–C** reprinted by permission from: Nature Neuroscience, Cruz Hernandez (11) ^©^2019 Springer Nature; **(D,E)** reproduced from Bennett et al. (33), by rights granted under a Creative Commons BY-NC-ND license).

A recent study using *in vivo* two photon microscopy in transgenic mice with excessive amyloid deposition demonstrated that an increased number of cortical capillaries had stalled blood flow due to neutrophils adhered to vessel wall (11) (Figures 4A–C). These stalls have also been found to correlate with amyloid deposition and, targeting neutrophil adhesion improved blood flow and cognitive performance (11). Another longitudinal study tracked obstructed capillaries and identified a VEGF-dependent pruning mechanism with regression of endothelial cells, causing uncompensated capillary loss (206). Perivascular macrophages, in response to amyloid-beta, can release reactive oxygen species that can aggravate microcirculatory dysfunction (207). Mouse models of increased hyperphosphorylated tau expression revealed capillary constrictions surrounded by swollen astrocyte processes (208), formation of abnormal spiraling capillary morphologies with impaired blood cell flux (33) (Figures 4D,E).

Transgenic mice with pericyte deficiency have been shown to have impaired capillary perfusion and neurovascular coupling, reduced oxygen supply, blood-brain barrier disruption, white matter degeneration and, importantly, progressive AD-like pathology (including progressive tauopathy and neuronal loss), resulting in accelerated cognitive decline (31, 100, 209–211). A recent study in AD patients showed reduced levels of platelet-derived growth factor-B, a pericyte marker, in precuneus, a cortical area affected in AD (101). We do not know exactly how pericytes degenerate progressively in those conditions, but there are some clues. For example, external introduction or overexpression of amyloid-β can damage pericytes directly (209, 212, 213) and conversely, pericytes can themselves be a zone of production of amyloid-β-associated proteins (214). Hypertension, dyslipidemia, and ApoE4 isoforms, all independent risk factors for AD, can also impair pericyte structure and function, that can potentially initiate a neurodegenerative phenotype (215–220). Pathogenic hyperphosphorylated tau, on the other hand, promotes BBB injury directly (221); the possibility that this injury may be related to pericyte damage, however, remains to be investigated.

### Other Chronic Neurodegenerative Diseases

Accumulating evidence suggests that several other progressive neurodegenerative conditions may also involve primary microcirculatory pathologies, Like AD and CTE as discussed above, other tauopathies such as corticobasal degeneration (CBD), progressive supranuclear palsy (PSP) and amyotrophic lateral sclerosis (ALS) exhibit possible microvascular involvement (222).

In Parkinson's disease, both experimental and clinical data suggest microvascular involvement. Capillary loss and abnormal fragmented capillaries or endothelial clusters can be detected in substantia nigra, cerebral cortex and brain stem in pathological brain samples (223). There are also compensatory new vessel formations, which are possibly immature and leaky. In animal models produced by MPTP treatment, decreased capillary density or formation of abnormal endothelial clusters have been reported as observed in autopsy brains (224).

CBD and PSP are two atypical and rapidly progressive forms of parkinsonism. In postmortem brains of CBD and PSP patients, prominent accumulation of hyperphosphorylated tau in close proximity to vasculature, astrocytic proliferation, and hypertrophy as well as reduced capillary diameter have been detected (225, 226). These findings are difficult to test experimentally since there are no established animal models of these conditions. There are transgenic mouse models, however, available for another tauopathy, ALS, with mutations in superoxide-dismutase (SOD1) (227), tar DNA binding protein (TARDBP/TDP43) (228), fused in sarcoma (FUS) (229), or C9orf72 (230) genes. SOD1 mouse model exhibits progressive decrease in the anterior gray matter of the spinal cord, beginning from the presymptomatic stage before neuronal degeneration, and is accompanied by a reduction in capillary diameter and density as well as slowing down of RBC flow (231). The flow-metabolism coupling is also disrupted as glucose utilization in contrast to the oxygen availability is increased especially in early stages. These findings become more profound as the disease progresses (232). At present, there is no reported data on microvascular features of mice with other ALS-related mutations, however, future studies might provide deeper insight. In ALS patients, the cerebral blood flow is decreased especially in the prefrontal cortex (233, 234) and a clinical picture of frontotemporal dementia can accompany ALS (235). In ALS patients, the blood-spinal cord barrier is also damaged and extravasated hemoglobin and hemosiderin deposits can be detected in perivascular areas (236). In ALS transgenic mice, there is data showing capillary pericyte degeneration and blood-spinal cord barrier leakage, which can precede motor neuron degeneration (231, 237).

Finally in epilepsy, a group of disorders characterized with chronic repetitive seizures, progressive neurodegeneration can occur over time and also a single prolonged seizure can initiate such degeneration (238, 239). This has previously been attributed to glutamate-induced excitotoxicity (240). Since there is usually increased blood flow in seizure foci (241–243), one would not readily suspect from impaired oxygenation as a possible cause of neuronal loss. But as discussed above, capillary dysfunction may occur in the setting of increased cerebral blood flow (38). An elegant study with *in vivo* two-photon microscopy, however, identified individual capillaries with pericyte contractions and accompanying stalls in blood flow in hippocampus of epileptic mice during seizures and those abnormal capillary vasodynamics created an environment with irregular capillary flow and possibly microscopic hypoxia, which was associated with neurodegeneration in close proximity of constricted capillaries (244).

## Possible Therapeutic Approaches to Improve Capillary Flow and Oxygenation

The above-discussed findings strongly suggest that, to slow down or to reverse the acute and chronic neurodegeneration, the microcirculatory structures and dynamics must also be taken into account. Interventions to improve the microcirculation need to have specific targets to homogenize capillary flow and optimize oxygen and glucose extraction that would meet the metabolic needs at all times. These approaches should complement interventions for the macrovessels. Any attempt to increase the cerebral blood flow without correcting microcirculatory disturbances may aggravate heterogeneous shunting and paradoxically worsen oxygen extraction (38).

### Reactive Oxygen Species and Pericyte Contraction

Various scavengers can target reactive oxygen species that are responsible for vasospasms as well as capillary pericyte contraction and endothelial dysfunction. One of these, N-tert-Butyl-α-phenylnitrone (PBN), has been shown to improve microcirculation in a mouse model of cerebral ischemia, improving tissue injury and functional outcome (245). Since NO depletion may be contributory to capillary constriction, nitric oxide inhalation as well as nitrite infusion have been suggested to improve microcirculation and tissue oxygenation in various models of neurovascular disorders (246–252). Like nitric oxide, adenosine is involved in active physiological regulation of microvascular diameter and perfusion (58, 253). Adenosine is endogenously released as a metabolic end product of cerebral activity and external adenosine administration increases capillary diameter by relaxing pericytes in both myocardial and cerebral microcirculation, under ischemic conditions (99, 254–256). A major problem with nitric oxide donors and adenosine-based therapies is their systemic side effects, including systemic cardiovascular and respiratory changes (257–260). Novel strategies that allow targeted cerebral delivery of such potent drugs, like squalenoyl adenosine nanoassemblies (99), or nanoparticulated nitric oxide donors (261), and also inhaled nitric oxide that does not have systemic cardiovascular side effects, can be highly useful to promote clinical translation of this pharmacologic potential.

Capillary pericytes constrict in response to adrenergic receptor activation and with endothelin, therefore their antagonists could be considered as potential therapeutic targets (51, 262). Pericyte constriction is calcium-dependent as it involves actin machinery (30). Voltage-gated calcium channel blockers have been shown to decrease pericyte constrictions and also pericyte death, improving capillary transit time homogenization (262, 263). Ca^+2^-activated chloride channels can also potentiate pericyte depolarization and constriction and can be another target (262, 264). Rho-kinase pathway inhibition can reverse abnormal pericyte contractions (265, 266) and this approach has been found to be neuroprotective in mouse models of cerebral ischemia (267–269).

### Glycocalyx Integrity

Shedding of glycocalyx can impair homogeneities in RBC flux and increase endothelial-leukocyte adhesion. Glycocalyx has been protected by anti-thrombin administration in a rat sepsis model and this treatment decreased leukocyte adhesion, improving microcirculatory blood flow in mesentery (270, 271). Avoiding hyperglycemia is also beneficial for glycocalyx integrity (271). Similarly, a hydroxyethyl starch improved lung microcirculation by preventing experimental glycocalyx degradation by heparinase (90). Therefore, restoring glycocalyx integrity can be another potential treatment approach.

### Blood Viscosity and Hematology

Since increased leukocyte count, activation, and increase in hematocrit (either due to higher RBC number or dehydration) increase capillary clogging, stalls and heterogeneity, blood viscosity, and endothelial adhesion could be one of the possible targets. Phosphodiesterase inhibitors like pentoxifylline increase RBC flexibility and decrease blood viscosity (272, 273) and they can indeed improve microcirculatory profile while their direct effects on augmenting NO activity might be another contributor to this (274). Prevention of dehydration and concomitant infections could also be beneficial for a regular and optimal capillary flow (38). These two factors are among the most common triggers of delirium and cognitive fluctuations in elderly patients with dementia (275).

Decreasing leukocyte count has been reported to provide a beneficial outcome in animal models of cerebral ischemia (276). A monoclonal antibody against Ly6G surface proteins on neutrophils has improved microcirculation by reversing neutrophil-mediated capillary stalls in mice, increased blood flow and behavioral scores acutely in mice, an effect attributed to modulation of neutrophil adhesion in capillaries (11). The same antibody has been found to be beneficial in mice with subarachnoid hemorrhage (277) and after stroke in hyperlipidemic mice (278). The exact physiological role and endogenous receptor of Ly6G is currently unknown (279) but these experimental data require further investigations.

Other innovative approaches to modulate rheological parameters of blood cells are continuously being developed. Drag-reducing polymers improved flexibility of RBCs, decreasing cell stasis in capillaries, and plasma separation in capillary branching points, improving capillary flow heterogeneity in cerebral and myocardial ischemia models (280–282). Uptake of plasma-derived extracellular vesicles by RBCs have recently been shown to increase their deformability (283, 284).

## Conclusion

Our review can help the recognition that many microvascular pathogenetic mechanisms are shared across a variety of acute and chronic neurological conditions and these mechanisms are not simply by-standers but are actually role-players, emerging in very early phases of neurodegenerative conditions. With advancement of optical microscopy, MRI-based imaging tools having higher spatiotemporal resolution as well as computer models, the physiology and vulnerabilities of the microcirculation will be better elucidated. Investigators in the field should not be surprised to see the introduction of previously unknown details, which will eventually lead to much better neuroprotectants for many neurological disorders within the next decades.

## Author Contributions

ŞE drafted the manuscript and prepared the figures. TD drafted and critically revised the manuscript.

### Conflict of Interest Statement

The authors declare that the research was conducted in the absence of any commercial or financial relationships that could be construed as a potential conflict of interest.

## References

[1] Bosetti F Galis ZS Bynoe MS Charette M Cipolla MJ Del Zoppo GJ. “Small blood vessels: big health problems?”: scientific recommendations of the national institutes of health workshop. J Am Heart Assoc. (2016) 5:e004389. 10.1161/JAHA.116.004389 27815267 PMC5210346

[2] Sakadžić S Mandeville ET Gagnon L Musacchia JJ Yaseen MA Yucel MA. Large arteriolar component of oxygen delivery implies a safe margin of oxygen supply to cerebral tissue. Nat Commun. (2014) 5:5734. 10.1038/ncomms6734 25483924 PMC4260810

[3] Meyer EP Ulmann-Schuler A Staufenbiel M Krucker T. Altered morphology and 3D architecture of brain vasculature in a mouse model for Alzheimer's disease. Proc Natl Acad Sci USA. (2008) 105:3587–92. 10.1073/pnas.0709788105 18305170 PMC2265182

[4] Mabuchi T Lucero J Feng A Koziol JA del Zoppo GJ. Focal cerebral ischemia preferentially affects neurons distant from their neighboring microvessels. J Cereb Blood Flow Metab. (2005) 25:257–66. 10.1038/sj.jcbfm.9600027 15678127

[5] Lovick TA Brown LA Key BJ. Neurovascular relationships in hippocampal slices: physiological and anatomical studies of mechanisms underlying flow-metabolism coupling in intraparenchymal microvessels. Neuroscience. (1999) 92:47–60. 10.1016/S0306-4522(98)00737-4 10392829

[6] Kanno I Masamoto K Bridging macroscopic and microscopic methods for the measurements of cerebral blood flow: toward finding the determinants in maintaining the CBF homeostasis. In: New Horizons in Neurovascular Coupling: A Bridge Between Brain Circulation and Neural Plasticity. Vol. 255 1st ed. (Amsterdam: Elsevier B.V.) (2016). 10.1016/bs.pbr.2016.03.002 27130412

[7] Raichle ME Mintun MA. Brain Work and Brain Imaging. Annu Rev Neurosci. (2006) 29:449–76. 10.1146/annurev.neuro.29.051605.112819 16776593

[8] Østergaard L Jørgensen MB Knudsen GM. Low on energy? An energy supply-demand perspective on stress and depression. Neurosci Biobehav Rev. (2018) 94:248–70. 10.1016/j.neubiorev.2018.08.007 30145282

[9] Iadecola C Nedergaard M. Glial regulation of the cerebral microvasculature. Nat Neurosci. (2007) 10:1369–76. 10.1038/nn2003 17965657

[10] Erdener SE Tang J Sajjadi A Kiliç K Kura S Schaffer CB. Spatio-temporal dynamics of cerebral capillary segments with stalling red blood cells. J Cereb Blood Flow Metab. (2019) 39:886–900. 10.1177/0271678X17743877 29168661 PMC6501506

[11] Cruz Hernández JC Bracko O Kersbergen CJ Muse V Haft-Javaherian M Berg M Neutrophil adhesion in brain capillaries reduces cortical blood flow and impairs memory function in Alzheimer's disease mouse models. Nat Neurosci. (2019) 22:413–20. 10.1038/s41593-018-0329-4 30742116 PMC6508667

[12] Shih AY Driscoll JD Drew PJ Nishimura N Schaffer CB Kleinfeld D. Two-photon microscopy as a tool to study blood flow and neurovascular coupling in the rodent brain. J Cereb Blood Flow Metab. (2012) 32:1277–309. 10.1038/jcbfm.2011.196 22293983 PMC3390800

[13] Kisler K Lazic D Sweeney MD Plunkett S El Khatib M Vinogradov SA. *In vivo* imaging and analysis of cerebrovascular hemodynamic responses and tissue oxygenation in the mouse brain. Nat Protoc. (2018) 13:1377–402. 10.1038/nprot.2018.034 29844521 PMC6402338

[14] Kleinfeld D Mitra PP Helmchen F Denk W. Fluctuations and stimulus-induced changes in blood flow observed in individual capillaries in layers 2 through 4 of rat neocortex. Proc Natl Acad Sci USA. (1998) 95:15741–6. 10.1073/pnas.95.26.15741 9861040 PMC28114

[15] Yaseen MA Srinivasan VJ SakadŽić S Radhakrishnan H Gorczynska I Wu W. Microvascular oxygen tension and flow measurements in rodent cerebral cortex during baseline conditions and functional activation. J Cereb Blood Flow Metab. (2011) 31:1051–63. 10.1038/jcbfm.2010.227 21179069 PMC3070982

[16] Devor A Sakadzic S Saisan PA Yaseen MA Roussakis E Srinivasan VJ “Overshoot” of O2 is required to maintain baseline tissue oxygenation at locations distal to blood vessels. J Neurosci. (2011) 31:13676–81. 10.1523/JNEUROSCI.1968-11.2011 21940458 PMC3188944

[17] SakadŽic S Yaseen MA Jaswal R Roussakis E Dale AM Buxton RB. Two-photon microscopy measurement of cerebral metabolic rate of oxygen using periarteriolar oxygen concentration gradients. Neurophotonics. (2016) 3:045005. 10.1117/1.NPh.3.4.045005 27774493 PMC5066455

[18] Sakadzić S Yuan S Dilekoz E Ruvinskaya S Vinogradov SA Ayata C. Simultaneous imaging of cerebral partial pressure of oxygen and blood flow during functional activation and cortical spreading depression. Appl Opt. (2009) 48:D169–77. 10.1364/AO.48.00D169 19340106 PMC2692697

[19] Sinks LE Robbins GP Roussakis E Troxler T Hammer DA Vinogradov SA. Two-photon microscopy of oxygen: polymersomes as probe carrier vehicles. J Phys Chem B. (2010) 114:14373–82. 10.1021/jp100353v 20462225 PMC2939231

[20] Lecoq J Parpaleix A Roussakis E Ducros M Houssen YG Vinogradov SA. Simultaneous two-photon imaging of oxygen and blood flow in deep cerebral vessels. Nat Med. (2011) 17:893–8. 10.1038/nm.2394 21642977 PMC3291110

[21] Esipova TV Barrett MJP Erlebach E Masunov AE Weber B Vinogradov SA. Oxyphor 2P: a high-performance probe for deep-tissue longitudinal oxygen imaging. Cell Metab. (2019) 29:736–44. 10.1016/j.cmet.2018.12.022 30686745 PMC6402963

[22] Devor A SakadŽić S Srinivasan VJ Yaseen MA Nizar K Saisan PA Tian P. Frontiers in optical imaging of cerebral blood flow and metabolism. J Cereb Blood Flow Metab. (2012) 32:1259–76. 10.1038/jcbfm.2011.195 22252238 PMC3390808

[23] Srinivasan VJ SakadŽić S Gorczynska I Ruvinskaya S Wu W Fujimoto JG. Quantitative cerebral blood flow with optical coherence tomography. Opt Express. (2010) 18:2477. 10.1364/OE.18.002477 20174075 PMC2837842

[24] Tang J Erdener SE Fu B Boas DA. Capillary red blood cell velocimetry by phase-resolved optical coherence tomography. Opt Lett. (2017) 42:3976. 10.1364/OL.42.003976 28957175 PMC5972360

[25] Lee J Wu W Jiang JY Zhu B Boas DA. Dynamic light scattering optical coherence tomography. Opt Express. (2012) 20:22262–77. 10.1364/OE.20.022262 23037374 PMC3601731

[26] Tang J Erdener SE Li B Fu B Sakadzic S Carp SA. Shear-induced diffusion of red blood cells measured with dynamic light scattering-optical coherence tomography. J Biophotonics. (2018) 11:1–10. 10.1002/jbio.201700070 28700129 PMC5766442

[27] Mouridsen K Hansen MB Østergaard L Jespersen SN. Reliable estimation of capillary transit time distributions using DSC-MRI. J Cereb Blood Flow Metab. (2014) 34:1511–21. 10.1038/jcbfm.2014.111 24938401 PMC4158667

[28] Yemisci M Gursoy-Ozdemir Y Vural A Can A Topalkara K Dalkara T. Pericyte contraction induced by oxidative-nitrative stress impairs capillary reflow despite successful opening of an occluded cerebral artery. Nat Med. (2009) 15:1031–7. 10.1038/nm.2022 19718040

[29] Iadecola C. The neurovascular unit coming of age: a journey through neurovascular coupling in health and disease. Neuron. (2017) 96:17–42. 10.1016/j.neuron.2017.07.030 28957666 PMC5657612

[30] Hall CN Reynell C Gesslein B Hamilton NB Mishra A Sutherland BA. Capillary pericytes regulate cerebral blood flow in health and disease. Nature. (2014) 508:55–60. 10.1038/nature13165 24670647 PMC3976267

[31] Kisler K Nelson AR Rege SV Ramanathan A Wang Y Ahuja A. Pericyte degeneration leads to neurovascular uncoupling and limits oxygen supply to brain. Nat Neurosci. (2017) 20:406–16. 10.1038/nn.4489 28135240 PMC5323291

[32] Lee J Wu W Boas DA. Early capillary flux homogenization in response to neural activation. J Cereb Blood Flow Metab. (2016) 36:375–80. 10.1177/0271678X15605851 26661145 PMC4759663

[33] Bennett RE Robbins AB Hu M Cao X Betensky RA Clark T. Tau induces blood vessel abnormalities and angiogenesis-related gene expression in P301L transgenic mice and human Alzheimer's disease. Proc Natl Acad Sci USA. (2018) 115:E1289–98. 10.1073/pnas.1710329115 29358399 PMC5819390

[34] Gagnon L SakadŽić S Lesage F Pouliot P Dale AM Devor A. Validation and optimization of hypercapnic-calibrated fMRI from oxygen-sensitive two-photon microscopy. Philos Trans R Soc B Biol Sci. (2016) 371:20150359. 10.1098/rstb.2015.0359 27574311 PMC5003859

[35] Cheng X Berman AJL Polimeni JR Buxton RB Gagnon L Devor A. Dependence of the MR signal on the magnetic susceptibility of blood studied with models based on real microvascular networks. Magn Reson Med. (2019) 81:3865–74. 10.1002/mrm.27660 30659643 PMC6435380

[36] Østergaard L Jespersen SN Mouridsen K Mikkelsen IK Jonsdottír KÝ Tietze A. The role of the cerebral capillaries in acute ischemic stroke: the extended penumbra model. J Cereb Blood Flow Metab. (2013) 33:635–48. 10.1038/jcbfm.2013.18 23443173 PMC3652700

[37] Jespersen SN Østergaard L. The roles of cerebral blood flow, capillary transit time heterogeneity, and oxygen tension in brain oxygenation and metabolism. J Cereb Blood Flow Metab. (2012) 32:264–77. 10.1038/jcbfm.2011.153 22044867 PMC3272609

[38] Østergaard L Engedal TS Moreton F Hansen MB Wardlaw JM Dalkara T. Cerebral small vessel disease: capillary pathways to stroke and cognitive decline. J Cereb Blood Flow Metab. (2016) 36:302–25. 10.1177/0271678X15606723 26661176 PMC4759673

[39] Østergaard L Aamand R Karabegovic S Tietze A Blicher JU Mikkelsen IK. The role of the microcirculation in delayed cerebral ischemia and chronic degenerative changes after subarachnoid hemorrhage. J Cereb Blood Flow Metab. (2013) 33:1825–37. 10.1038/jcbfm.2013.173 24064495 PMC3851911

[40] Balogh P Bagchi P Analysis of red blood cell partitioning at bifurcations in simulated microvascular networks. Phys Fluids. (2018) 30:051902 10.1063/1.5024783

[41] Santisakultarm TP Paduano CQ Stokol T Southard TL Nishimura N Skoda RC. Stalled cerebral capillary blood flow in mouse models of essential thrombocythemia and polycythemia vera revealed by *in vivo* two-photon imaging. J Thromb Haemost. (2014) 12:2120–30. 10.1111/jth.12738 25263265

[42] Kataoka H Kim SW Plesnila N. Leukocyte-endothelium interactions during permanent focal cerebral ischemia in mice. J Cereb Blood Flow Metab. (2004) 24:668–76. 10.1097/01.WCB.0000117812.35136.5B 15181374

[43] Østergaard L Jespersen SN Engedahl T Jiménez EG Ashkanian M Hansen MB. Capillary dysfunction: its detection and causative role in dementias and stroke. Curr Neurol Neurosci Rep. (2015) 15:557. 10.1007/s11910-015-0557-x 25956993 PMC4441906

[44] Angleys H Østergaard L Jespersen SN. The effects of capillary transit time heterogeneity (CTH) on brain oxygenation. J Cereb Blood Flow Metab. (2015) 35:806–17. 10.1038/jcbfm.2014.254 25669911 PMC4420854

[45] Daneman R Prat A. The blood–brain barrier. Cold Spring Harb Perspect Biol. (2015) 7:a020412. 10.1101/cshperspect.a020412 25561720 PMC4292164

[46] Gu Y Zheng G Xu M Li Y Chen X Zhu W. Caveolin-1 regulates nitric oxide-mediated matrix metalloproteinases activity and blood-brain barrier permeability in focal cerebral ischemia and reperfusion injury. J Neurochem. (2012) 120:147–56. 10.1111/j.1471-4159.2011.07542.x 22007835

[47] Oldendorf WH Cornford ME Brown WJ. The large apparent work capability of the blood-brain barrier: a study of the mitochondrial content of capillary endothelial cells in brain and other tissues of the rat. Ann Neurol. (1977) 1:409–17. 10.1002/ana.410010502 617259

[48] Friis ML Paulson OB Hertz MM. Carbon dioxide permeability of the blood-brain barrier in man: The effect of acetazolamide. Microvasc Res. (1980) 20:71–80. 10.1016/0026-2862(80)90020-5 6774205

[49] Srinivasan VJ Yu E Radhakrishnan H Can A Climov M Leahy C. Micro-heterogeneity of flow in a mouse model of chronic cerebral hypoperfusion revealed by longitudinal doppler optical coherence tomography and angiography. J Cereb Blood Flow Metab. (2015) 35:1552–60. 10.1038/jcbfm.2015.175 26243708 PMC4640323

[50] Armulik A Abramsson A Betsholtz C. Endothelial/pericyte interactions. Circ Res. (2005) 97:512–23. 10.1161/01.RES.0000182903.16652.d7 16166562

[51] Peppiatt CM Howarth C Mobbs P Attwell D. Bidirectional control of CNS capillary diameter by pericytes. Nature. (2006) 443:700–4. 10.1038/nature05193 17036005 PMC1761848

[52] Risau W Wolburg H. Development of the blood-brain barrier. Trends Neurosci. (1990) 13:174–8. 10.1016/0166-2236(90)90043-A 1693235

[53] Dore-Duffy P LaManna JC. Physiologic angiodynamics in the brain. Antioxid Redox Signal. (2007) 9:1363–71. 10.1089/ars.2007.1713 17627476

[54] Thomas WE. Brain macrophages: on the role of pericytes and perivascular cells. Brain Res Brain Res Rev. (1999) 31:42–57. 10.1016/S0165-0173(99)00024-7 10611494

[55] Armulik A Genové G Mäe M Nisancioglu MH Wallgard E Niaudet C. Pericytes regulate the blood-brain barrier. Nature. (2010) 468:557–61. 10.1038/nature09522 20944627

[56] ElAli A Thériault P Rivest S. The role of pericytes in neurovascular unit remodeling in brain disorders. Int J Mol Sci. (2014) 15:6453–74. 10.3390/ijms15046453 24743889 PMC4013640

[57] Yang S Jin H Zhu Y Wan Y Opoku EN Zhu L. Diverse functions and mechanisms of pericytes in ischemic stroke. Curr Neuropharmacol. (2017) 15:892–905. 10.2174/1570159X15666170112170226 28088914 PMC5652032

[58] Attwell D Buchan AM Charpak S Lauritzen M MacVicar BA Newman EA. Glial and neuronal control of brain blood flow. Nature. (2010) 468:232–43. 10.1038/nature09613 21068832 PMC3206737

[59] Jakovcevic D Harder DR Role of astrocytes in matching blood flow to neuronal activity. In: Schatten GP, editor. Current Topics in Developmental Biology. Vol. 79. (San Diego, CA: Academic Press) (2007). p. 75–97. 10.1016/S0070-2153(06)79004-4 17498548

[60] Magistretti PJ Allaman I. Lactate in the brain: from metabolic end-product to signalling molecule. Nat Rev Neurosci. (2018) 19:235–49. 10.1038/nrn.2018.19 29515192

[61] Bennett MVL Contreras JE Bukauskas FF Sáez JC. New roles for astrocytes: gap junction hemichannels have something to communicate. Trends Neurosci. (2003) 26:610–7. 10.1016/j.tins.2003.09.008 14585601 PMC3694339

[62] Parys B Côté A Gallo V De Koninck P Sík A. Intercellular calcium signaling between astrocytes and oligodendrocytes via gap junctions in culture. Neuroscience. (2010) 167:1032–43. 10.1016/j.neuroscience.2010.03.004 20211698

[63] Gu X Chen W Volkow ND Koretsky AP Du C Pan Y. Synchronized astrocytic Ca2+responses in neurovascular coupling during somatosensory stimulation and for the resting state. Cell Rep. (2018) 23:3878–90. 10.1016/j.celrep.2018.05.091 29949771 PMC7469112

[64] Segal SS Duling BR. Flow control among microvessels coordinated by intercellular conduction. Science. (1986) 234:868–70. 10.1126/science.3775368 3775368

[65] Isakson BE Damon DN Day KH Liao Y Duling BR. Connexin40 and connexin43 in mouse aortic endothelium: evidence for coordinated regulation. Am J Physiol Heart Circ Physiol. (2006) 290:H1199–205. 10.1152/ajpheart.00945.2005 16284228

[66] Little TL Beyer EC Duling BR. Connexin 43 and connexin 40 gap junctional proteins are present in arteriolar smooth muscle and endothelium *in vivo*. Am J Physiol Circ Physiol. (1995) 268:H729–39. 10.1152/ajpheart.1995.268.2.H729 7864199

[67] Pries AR Höpfner M le Noble F Dewhirst MW Secomb TW. The shunt problem: control of functional shunting in normal and tumour vasculature. Nat Rev Cancer. (2010) 10:587–93. 10.1038/nrc2895 20631803 PMC3109666

[68] Isakson BE Kronke G Kadl A Leitinger N Duling BR. Oxidized phospholipids alter vascular connexin expression, phosphorylation, and heterocellular communication. Arterioscler Thromb Vasc Biol. (2006) 26:2216–21. 10.1161/01.ATV.0000237608.19055.53 16857951

[69] Uhlirova H Kiliç K Tian P Thunemann M Desjardins M Saisan PA. Cell type specificity of neurovascular coupling in cerebral cortex. Elife. (2016) 5:14315. 10.7554/eLife.14315 27244241 PMC4933561

[70] Anenberg E Chan AW Xie Y LeDue JM Murphy TH. Optogenetic stimulation of GABA neurons can decrease local neuronal activity while increasing cortical blood flow. J Cereb Blood Flow Metab. (2015) 35:1579–86. 10.1038/jcbfm.2015.140 26082013 PMC4640302

[71] Cauli B Tong X-K Rancillac A Serluca N Lambolez B Rossier J. Cortical GABA interneurons in neurovascular coupling: relays for subcortical vasoactive pathways. J Neurosci. (2004) 24:8940–9. 10.1523/JNEUROSCI.3065-04.2004 15483113 PMC6730057

[72] Du W Stern JE Filosa JA. Neuronal-derived nitric oxide and somatodendritically released vasopressin regulate neurovascular coupling in the rat hypothalamic supraoptic nucleus. J Neurosci. (2015) 35:5330–41. 10.1523/JNEUROSCI.3674-14.2015 25834057 PMC4381004

[73] Sprague RS Stephenson AH Ellsworth ML Red not dead: signaling in and from erythrocytes. Trends Endocrinol Metab. (2007) 18:350–5. 10.1016/j.tem.2007.08.008 17959385

[74] Zhang H Shen Z Hogan B Barakat AI Misbah C. ATP release by red blood cells under flow: model and simulations. Biophys J. (2018) 115:2218–29. 10.1016/j.bpj.2018.09.033 30447988 PMC6289826

[75] Marginedas-Freixa I Alvarez CL Moras M Leal Denis MF Hattab C. Human erythrocytes release ATP by a novel pathway involving VDAC oligomerization independent of pannexin-1. Sci Rep. (2018) 8:11384. 10.1038/s41598-018-29885-7 30061676 PMC6065367

[76] Belcik JT Davidson BP Xie A Wu MD Yadava M Qi Y. Augmentation of muscle blood flow by ultrasound cavitation is mediated by ATP and purinergic signaling. Circulation. (2017) 135:1240–52. 10.1161/CIRCULATIONAHA.116.024826 28174191 PMC5373943

[77] Sridharan M Adderley SP Bowles EA Egan TM Stephenson AH Ellsworth ML Sprague RS. Pannexin 1 is the conduit for low oxygen tension-induced ATP release from human erythrocytes. Am J Physiol Heart Circ Physiol. (2010) 299:H1146–52. 10.1152/ajpheart.00301.2010 20622111 PMC2957350

[78] Takano T Tian G-F Peng W Lou N Libionka W Han X. Astrocyte-mediated control of cerebral blood flow. Nat Neurosci. (2006) 9:260–7. 10.1038/nn1623 16388306

[79] Astrup J Siesjö BK Symon L. Thresholds in cerebral ischemia - the ischemic penumbra. Stroke. (1981) 12:723–5. 10.1161/01.STR.12.6.723 6272455

[80] Iihara K Okawa M Hishikawa T Yamada N Fukushima K Iida H. Slowly progressive neuronal death associated with postischemic hyperperfusion in cortical laminar necrosis after high-flow bypass for a carotid intracavernous aneurysm. J Neurosurg. (2010) 112:1254–9. 10.3171/2009.9.JNS09345 19877803

[81] Wegener S Artmann J Luft AR Buxton RB Weller M Wong EC. The time of maximum post-ischemic hyperperfusion indicates infarct growth following transient experimental ischemia. PLoS ONE. (2013) 8:e65322. 10.1371/journal.pone.0065322 23741488 PMC3669346

[82] Engedal TS Hjort N Hougaard KD Simonsen CZ Andersen G Mikkelsen IK. Transit time homogenization in ischemic stroke – A novel biomarker of penumbral microvascular failure? J Cereb Blood Flow Metab. (2018) 38:2006–20. 10.1177/0271678X17721666 28758524 PMC6259320

[83] Park CS Payne SJ. Modelling the effects of cerebral microvasculature morphology on oxygen transport. Med Eng Phys. (2016) 38:41–7. 10.1016/j.medengphy.2015.09.004 26499366 PMC4751405

[84] Gagnon L Smith AF Boas DA Devor A Secomb TW SakadŽić S. Modeling of cerebral oxygen transport based on *in vivo* microscopic imaging of microvascular network structure, blood flow, and oxygenation. Front Comput Neurosci. (2016) 10:1–20. 10.3389/fncom.2016.00082 27630556 PMC5006088

[85] Lücker A Secomb TW Barrett MJP Weber B Jenny P. The relation between capillary transit times and hemoglobin saturation heterogeneity. part 2: capillary networks. Front Physiol. (2018) 9:1296. 10.3389/fphys.2018.01296 30298017 PMC6160581

[86] Eskildsen SF Gyldensted L Nagenthiraja K Nielsen RB Hansen MB Dalby RB. Increased cortical capillary transit time heterogeneity in Alzheimer's disease: a DSC-MRI perfusion study. Neurobiol Aging. (2017) 50:107–18. 10.1016/j.neurobiolaging.2016.11.004 27951412

[87] Secomb TW Hsu R Pries AR. A model for red blood cell motion in glycocalyx-lined capillaries. Am J Physiol. (1998) 274:H1016–22. 10.1152/ajpheart.1998.274.3.H1016 9530216

[88] Vink H Duling BR. Identification of distinct luminal domains for macromolecules, erythrocytes, and leukocytes within mammalian capillaries. Circ Res. (1996) 79:581–9. 10.1161/01.RES.79.3.581 8781491

[89] McClatchey PM Schafer M Hunter KS Reusch JEB. The endothelial glycocalyx promotes homogenous blood flow distribution within the microvasculature. Am J Physiol Heart Circ Physiol. (2016) 311:H168–76. 10.1152/ajpheart.00132.2016 27199117 PMC6189750

[90] Strunden MS Bornscheuer A Schuster A Kiefmann R Goetz AE Heckel K. Glycocalyx degradation causes microvascular perfusion failure in the *ex vivo* perfused mouse lung. Shock. (2012) 38:559–66. 10.1097/SHK.0b013e31826f2583 23042196

[91] Mas M A close look at the endothelium: its role in the regulation of vasomotor tone. Eur Urol Suppl. (2009) 8:48–57. 10.1016/j.eursup.2008.10.011

[92] Favero G Paganelli C Buffoli B Rodella LF Rezzani R. Endothelium and its alterations in cardiovascular diseases: life style intervention. Biomed Res Int. (2014) 2014:801896. 10.1155/2014/801896 24719887 PMC3955677

[93] Lefer AM. Nitric oxide: nature's naturally occurring leukocyte inhibitor. Circulation. (1997) 95:553–4. 10.1161/01.CIR.95.3.553 9024134

[94] Kubes P Suzuki M Granger DN. Nitric oxide: an endogenous modulator of leukocyte adhesion. Proc Natl Acad Sci USA. (1991) 88:4651–5. 10.1073/pnas.88.11.4651 1675786 PMC51723

[95] Cines DB Pollak ES Buck CA Loscalzo J Zimmerman GA McEver RP. Endothelial cells in physiology and in the pathophysiology of vascular disorders. Blood. (1998) 91:3527–61. 9572988

[96] Wang S Cao C Chen Z Bankaitis V Tzima E Sheibani N. Pericytes regulate vascular basement membrane remodeling and govern neutrophil extravasation during inflammation. PLoS ONE. (2012) 7:e45499. 10.1371/journal.pone.0045499 23029055 PMC3448630

[97] Schmid F Reichold J Weber B Jenny P. The impact of capillary dilation on the distribution of red blood cells in artificial networks. Am J Physiol Heart Circ Physiol. (2015) 308:H733–42. 10.1152/ajpheart.00335.2014 25617356

[98] Špiranec K Chen W Werner F Nikolaev VO Naruke T Koch F. Endothelial C-type natriuretic peptide acts on pericytes to regulate microcirculatory flow and blood pressure. Circulation. (2018) 138:494–508. 10.1161/CIRCULATIONAHA.117.033383 29626067

[99] Gaudin A Yemisci M Eroglu H Lepetre-Mouelhi S Turkoglu OF Dönmez-Demir B. Squalenoyl adenosine nanoparticles provide neuroprotection after stroke and spinal cord injury. Nat Nanotechnol. (2014) 9:1054–62. 10.1038/nnano.2014.274 25420034 PMC4351925

[100] Bell RD Winkler EA Sagare AP Singh I LaRue B Deane R. Pericytes control key neurovascular functions and neuronal phenotype in the adult brain and during brain aging. Neuron. (2010) 68:409–27. 10.1016/j.neuron.2010.09.043 21040844 PMC3056408

[101] Miners JS Schulz I Love S. Differing associations between Aβ accumulation, hypoperfusion, blood–brain barrier dysfunction and loss of PDGFRB pericyte marker in the precuneus and parietal white matter in Alzheimer's disease. J Cereb Blood Flow Metab. (2018) 38:103–15. 10.1177/0271678X17690761 28151041 PMC5757436

[102] Schultz N Brännström K Byman E Moussaud S Nielsen HM Olofsson A. Amyloid-beta 1-40 is associated with alterations in NG2+ pericyte population *ex vivo* and *in vitro*. Aging Cell. (2018) 17:e12728. 10.1111/acel.12728 29453790 PMC5946076

[103] Lin W Ma X Hao M Zhou H Yu X Shao N. Liraglutide attenuates the migration of retinal pericytes induced by advanced glycation end products. Peptides. (2018) 105:7–13. 10.1016/j.peptides.2018.05.003 29746877

[104] Reeves C Jardim AP Sisodiya SM Thom M Liu JYW. Spatiotemporal dynamics of PDGFRβ expression in pericytes and glial scar formation in penetrating brain injuries in adults. Neuropathol Appl Neurobiol. (2019). 10.1111/nan.12539. [Epub ahead of print]. 30636077 PMC6767497

[105] Dore-Duffy P Owen C Balabanov R Murphy S Beaumont T Rafols JA. Pericyte migration from the vascular wall in response to traumatic brain injury. Microvasc Res. (2000) 60:55–69. 10.1006/mvre.2000.2244 10873515

[106] Berthiaume AA Grant RI McDowell KP Underly RG Hartmann DA Levy M. Dynamic remodeling of pericytes *in vivo* maintains capillary coverage in the adult mouse brain. Cell Rep. (2018) 22:8–16. 10.1016/j.celrep.2017.12.016 29298435 PMC5782812

[107] Berthiaume AA Hartmann DA Majesky MW Bhat NR Shih AY. Pericyte structural remodeling in cerebrovascular health and homeostasis. Front Aging Neurosci. (2018) 10:210. 10.3389/fnagi.2018.00210 30065645 PMC6057109

[108] Mazzoni MC Schmid-Schonbein GW. Mechanisms and consequences of cell activation in the microcirculation. Cardiovasc Res. (1996) 32:709–19. 10.1016/S0008-6363(96)00146-0 8915189

[109] Barnes AJ Locke P Scudder PR Dormandy TL Dormandy JA Slack J. Is hyperviscosity a treatable component of diabetic microcirculatory disease? Lancet. (1977) 2:789–91. 10.1016/S0140-6736(77)90724-3 71601

[110] Mori E del Zoppo GJ Chambers JD Copeland BR Arfors KE. Inhibition of polymorphonuclear leukocyte adherence suppresses no- reflow after focal cerebral ischemia in baboons. Stroke. (1992) 23:712–8. 10.1161/01.STR.23.5.712 1579969

[111] del Zoppo GJ Schmid-Schönbein GW Mori E Copeland BR Chang CM. Polymorphonuclear leukocytes occlude capillaries following middle cerebral artery occlusion and reperfusion in baboons. Stroke. (1991) 22:1276–83. 10.1161/01.STR.22.10.1276 1926239

[112] Ritter LS Orozco JA Coull BM McDonagh PF Rosenblum WI Leukocyte accumulation and hemodynamic changes in the cerebral microcirculation during early reperfusion after stroke editorial comment. Stroke. (2000) 31:1153–61. 10.1161/01.STR.31.5.1153 10797180

[113] Clark WM Coull BM Briley DP Mainolfi E Rothlein R. Circulating intercellular adhesion molecule-1 levels and neutrophil adhesion in stroke. J Neuroimmunol. (1993) 44:123–5. 10.1016/0165-5728(93)90275-4 8098717

[114] Harris AG Skalak TC. Effects of leukocyte capillary plugging in skeletal muscle ischemia-reperfusion injury. Am J Physiol. (1996) 271:H2653–60. 10.1152/ajpheart.1996.271.6.H2653 8997328

[115] Theilen H Schröck H Kuschinsky W. Gross persistence of capillary plasma perfusion after middle cerebral artery occlusion in the rat brain. J Cereb Blood Flow Metab. (1994) 14:1055–61. 10.1038/jcbfm.1994.138 7929648

[116] Villringer A Them A Lindauer U Einhäupl K Dirnagl U. Capillary perfusion of the rat brain cortex. An *in vivo* confocal microscopy study. Circ Res. (1994) 75:55–62. 10.1161/01.RES.75.1.55 8013082

[117] Abounader R Vogel J Kuschinsky W. Patterns of capillary plasma perfusion in brains of conscious rats during normocapnia and hypercapnia. Circ Res. (1995) 76:120–6. 10.1161/01.RES.76.1.120 8001269

[118] Bookheimer SY Strojwas MH Cohen MS Saunders AM Pericak-Vance MA Mazziotta JC. Patterns of brain activation in people at risk for Alzheimer's disease. N Engl J Med. (2000) 343:450–6. 10.1056/NEJM200008173430701 10944562 PMC2831477

[119] Scarmeas N Habeck CG Stern Y Anderson KE. APOE genotype and cerebral blood flow in healthy young individuals. JAMA. (2003) 290:1581–2. 10.1001/jama.290.12.1581 14506116 PMC3026566

[120] Kim T Richard Jennings J Kim SG. Regional cerebral blood flow and arterial blood volume and their reactivity to hypercapnia in hypertensive and normotensive rats. J Cereb Blood Flow Metab. (2014) 34:408–14. 10.1038/jcbfm.2013.197 24252849 PMC3948115

[121] Angleys H Jespersen SN Østergaard L. The effects of capillary transit time heterogeneity on the BOLD signal. Hum Brain Mapp. (2018) 39:2329–52. 10.1002/hbm.23991 29498762 PMC6866377

[122] Brown WR. A review of string vessels or collapsed, empty basement membrane tubes. J Alzheimer Dis. (2010) 21:725–39. 10.3233/JAD-2010-100219 20634580 PMC3081641

[123] Hunter JM Kwan J Malek-Ahmadi M Maarouf CL Kokjohn TA Belden C. Morphological and pathological evolution of the brain microcirculation in aging and alzheimer's disease. PLoS ONE. (2012) 7:e36893. 10.1371/journal.pone.0036893 22615835 PMC3353981

[124] Tong X-K Hamel E. Simvastatin restored vascular reactivity, endothelial function and reduced string vessel pathology in a mouse model of cerebrovascular disease. J Cereb Blood Flow Metab. (2015) 35:512–20. 10.1038/jcbfm.2014.226 25564230 PMC4348394

[125] Pantoni L. Cerebral small vessel disease: from pathogenesis and clinical characteristics to therapeutic challenges. Lancet Neurol. (2010) 9:689–701. 10.1016/S1474-4422(10)70104-6 20610345

[126] Price TO Eranki V Banks WA Ercal N Shah GN. Topiramate treatment protects blood-brain barrier pericytes from hyperglycemia-induced oxidative damage in diabetic mice. Endocrinology. (2012) 153:362–72. 10.1210/en.2011-1638 22109883 PMC3249670

[127] McCuskey PA McCuskey RS. *In vivo* and electron microscopic study of the development of cerebral diabetic microangiography. Microcirc Endothelium Lymphatics. (1984) 1:221–44. 6546144

[128] Tagami M Nara Y Kubota A Fujino H Yamori Y. Ultrastructural changes in cerebral pericytes and astrocytes of stroke-prone spontaneously hypertensive rats. Stroke. (1990) 21:1064–71. 10.1161/01.STR.21.7.1064 2368108

[129] Junker U Jaggi C Bestetti G Rossi GL. Basement membrane of hypothalamus and cortex capillaries from normotensive and spontaneously hypertensive rats with streptozotocin-induced diabetes. Acta Neuropathol. (1985) 65:202–8. 10.1007/BF00686999 3976357

[130] Wood KC Hebbel RP Granger DN. Endothelial cell NADPH oxidase mediates the cerebral microvascular dysfunction in sickle cell transgenic mice. FASEB J. (2005) 19:989–91. 10.1096/fj.04-3218fje 15923406

[131] Gidday JM Park TS Shah AR Gonzales ER. Modulation of basal and postischemic leukocyte-endothelial adherence by nitric oxide. Stroke. (1998) 29:1423–30. 10.1161/01.STR.29.7.1423 9660399

[132] Fraticelli A Serrano CV Bochner BS Capogrossi MC Zweier JL. Hydrogen peroxide and superoxide modulate leukocyte adhesion molecule expression and leukocyte endothelial adhesion. Biochim Biophys Acta. (1996) 1310:251–9. 10.1016/0167-4889(95)00169-7 8599602

[133] Vink H Constantinescu AA Spaan JA. Oxidized lipoproteins degrade the endothelial surface layer : implications for platelet-endothelial cell adhesion. Circulation. (2000) 101:1500–2. 10.1161/01.CIR.101.13.1500 10747340

[134] Czarnowska E Karwatowska-Prokopczuk E. Ultrastructural demonstration of endothelial glycocalyx disruption in the reperfused rat heart. Involvement of oxygen free radicals. Basic Res Cardiol. (1995) 90:357–64. 10.1007/BF00788496 8585856

[135] Pantoni L Garcia JH. Pathogenesis of leukoaraiosis. Stroke. (1997) 28:652–59. 10.1161/01.STR.28.3.652 9056627

[136] Salat DH. Imaging small vessel-associated white matter changes in aging. Neuroscience. (2014) 276:174–86. 10.1016/j.neuroscience.2013.11.041 24316059 PMC4048333

[137] Pires PW Dams Ramos CM Matin N Dorrance AM. The effects of hypertension on the cerebral circulation. Am J Physiol Heart Circ Physiol. (2013) 304:H1598–614. 10.1152/ajpheart.00490.2012 23585139 PMC4280158

[138] Shi Y Wardlaw JM. Update on cerebral small vessel disease: a dynamic whole-brain disease. Stroke Vasc Neurol. (2016) 1:83–92. 10.1136/svn-2016-000035 28959468 PMC5435198

[139] Martinez-Quinones P McCarthy CG Watts SW Klee NS Komic A Calmasini FB Priviero F. Hypertension induced morphological and physiological changes in cells of the arterial wall. Am J Hypertens. (2018) 31:1067–78. 10.1093/ajh/hpy083 29788246 PMC6132119

[140] Umemura T Kawamura T Hotta N. Pathogenesis and neuroimaging of cerebral large and small vessel disease in type 2 diabetes: a possible link between cerebral and retinal microvascular abnormalities. J Diabetes Investig. (2017) 8:134–48. 10.1111/jdi.12545 27239779 PMC5334292

[141] Caplan LR. Diabetes and brain ischemia. Diabetes. (1996) 45(Suppl 3):S95–7. 10.2337/diab.45.3.S95 8674904

[142] Gursoy-Ozdemir Y Yemisci M Dalkara T. Microvascular protection is essential for successful neuroprotection in stroke. J Neurochem. (2012) 123:2–11. 10.1111/j.1471-4159.2012.07938.x 23050637

[143] Dalkara T Arsava EM. Can restoring incomplete microcirculatory reperfusion improve stroke outcome after thrombolysis? J Cereb Blood Flow Metab. (2012) 32:2091–9. 10.1038/jcbfm.2012.139 23047270 PMC3519416

[144] De Silva DA Fink JN Christensen S Ebinger M Bladin C Levi CR. Assessing reperfusion and recanalization as markers of clinical outcomes after intravenous thrombolysis in the echoplanar imaging thrombolytic evaluation trial (EPITHET). Stroke. (2009) 40:2872–4. 10.1161/STROKEAHA.108.543595 19478228

[145] Soares BP Tong E Hom J Cheng SC Bredno J Boussel L. Reperfusion is a more accurate predictor of follow-up infarct volume than recanalization: a proof of concept using CT in acute ischemic stroke patients. Stroke. (2010) 41:e34–40. 10.1161/STROKEAHA.109.568766 19910542 PMC2909663

[146] Liu S Shi H Liu W Furuichi T Timmins GS Liu KJ. Interstitial pO2 in ischemic penumbra and core are differentially affected following transient focal cerebral ischemia in rats. J Cereb Blood Flow Metab. (2004) 24:343–9. 10.1097/01.WCB.0000110047.43905.01 15091115

[147] Maestrini I Strbian D Gautier S Haapaniemi E Moulin S Sairanen T. Higher neutrophil counts before thrombolysis for cerebral ischemia predict worse outcomes. Neurology. (2015) 85:1408–16. 10.1212/WNL.0000000000002029 26362283 PMC4626239

[148] Zhang J Ren Q Song Y He M Zeng Y Liu Z Xu J. Prognostic role of neutrophil–lymphocyte ratio in patients with acute ischemic stroke. Medicine. (2017) 96:e8624. 10.1097/MD.0000000000008624 29137097 PMC5690790

[149] Fang YN Tong MS Sung PH Chen YL Chen CH Tsai NW. Higher neutrophil counts and neutrophil-to-lymphocyte ratio predict prognostic outcomes in patients after non-atrial fibrillation-caused ischemic stroke. Biomed J. (2017) 40:154–62. 10.1016/j.bj.2017.03.002 28651737 PMC6136280

[150] Wang H Zhang M Hao Y Zi W Yang D Zhou Z. Early prediction of poor outcome despite successful recanalization after endovascular treatment for anterior large vessel occlusion stroke. World Neurosurg. (2018) 115:e312–21. 10.1016/j.wneu.2018.04.042 29673825

[151] Elkind MS. Why now? Moving from stroke risk factors to stroke triggers. Curr Opin Neurol. (2007) 20:51–7. 10.1097/WCO.0b013e328012da75 17215689

[152] Davis S Donnan GA. Time is Penumbra: imaging, selection and outcome. The Johann jacob wepfer award 2014. Cerebrovasc Dis. (2014) 38:59–72. 10.1159/000365503 25227260

[153] Guadagno JV Jones PS Aigbirhio FI Wang D Fryer TD Day DJ. Selective neuronal loss in rescued penumbra relates to initial hypoperfusion. Brain. (2008) 131:2666–78. 10.1093/brain/awn175 18678564

[154] Baron JC Yamauchi H Fujioka M Endres M. Selective neuronal loss in ischemic stroke and cerebrovascular disease. J Cereb Blood Flow Metab. (2014) 34:2–18. 10.1038/jcbfm.2013.188 24192635 PMC3887360

[155] Phan TG Wright PM Markus R Howells DW Davis SM Donnan GA. Salvaging the ischaemic penumbra: more than just reperfusion? Clin Exp Pharmacol Physiol. (2002) 29:1–10. 10.1046/j.1440-1681.2002.03609.x 11917903

[156] Murphy BD Fox AJ Lee DH Sahlas DJ Black SE Hogan MJ. Identification of penumbra and infarct in acute ischemic stroke using computed tomography perfusion-derived blood flow and blood volume measurements. Stroke. (2006) 37:1771–7. 10.1161/01.STR.0000227243.96808.53 16763182

[157] Bordeleau M ElAli A Rivest S. Severe chronic cerebral hypoperfusion induces microglial dysfunction leading to memory loss in APPswe/PS1 mice. Oncotarget. (2016) 7:11864–80. 10.18632/oncotarget.7689 26918610 PMC4914254

[158] ElAli A Thériault P Préfontaine P Rivest S. Mild chronic cerebral hypoperfusion induces neurovascular dysfunction, triggering peripheral beta-amyloid brain entry and aggregation. Acta Neuropathol Commun. (2013) 1:75. 10.1186/2051-5960-1-75 24252187 PMC3843528

[159] Song J Nan D He Q Yang L Guo H. Astrocyte activation and capillary remodeling in modified bilateral common carotid artery occlusion mice. Microcirculation. (2017) 24:e12366. 10.1111/micc.12366 28261893

[160] Takasugi J Miwa K Watanabe Y Okazaki S Todo K Sasaki T. Cortical cerebral microinfarcts on 3T magnetic resonance imaging in patients with carotid artery stenosis. Stroke. (2019) 50:639–44. 10.1161/STROKEAHA.118.023781 30744544

[161] Mundiyanapurath S Ringleb PA Diatschuk S Hansen MB Mouridsen K Østergaard L. Capillary transit time heterogeneity is associated with modified rankin scale score at discharge in patients with bilateral high grade internal carotid artery stenosis. PLoS ONE. (2016) 11:e0158148. 10.1371/journal.pone.0158148 27336668 PMC4919050

[162] Crowley RW Medel R Dumont AS Ilodigwe D Kassell NF Mayer SA. Angiographic vasospasm is strongly correlated with cerebral infarction after subarachnoid hemorrhage. Stroke. (2011) 42:919–23. 10.1161/STROKEAHA.110.597005 21350201

[163] Macdonald RL Weir BK. A review of hemoglobin and the pathogenesis of cerebral vasospasm. Stroke. (1991) 22:971–82. 10.1161/01.STR.22.8.971 1866764

[164] Takeuchi K Miyata N Renic M Harder DR Roman RJ. Hemoglobin NO, and 20-HETE interactions in mediating cerebral vasoconstriction following SAH. Am J Physiol Regul Integr Comp Physiol. (2006) 290:R84–9. 10.1152/ajpregu.00445.2005 16166205

[165] Friedrich B Müller F Feiler S Schöller K Plesnila N. Experimental subarachnoid hemorrhage causes early and long-lasting microarterial constriction and microthrombosis: an *in-vivo* microscopy study. J Cereb Blood Flow Metab. (2012) 32:447–55. 10.1038/jcbfm.2011.154 22146194 PMC3293113

[166] Anzabi M Angleys H Aamand R Ardalan M Mouridsen K Rasmussen PM. Capillary flow disturbances after experimental subarachnoid hemorrhage: a contributor to delayed cerebral ischemia? Microcirculation. (2018) 26:e12516. 10.1111/micc.12516 30431201

[167] McConnell ED Wei HS Reitz KM Kang H Takano T Vates GE. Cerebral microcirculatory failure after subarachnoid hemorrhage is reversed by hyaluronidase. J Cereb Blood Flow Metab. (2016) 36:1537–52. 10.1177/0271678X15608389 26661183 PMC5012515

[168] Ishikawa M Kajimura M Morikawa T Tsukada K Tsuji T Kusaka G. Cortical microcirculatory disturbance in the super acute phase of subarachnoid hemorrhage - *In vivo* analysis using two-photon laser scanning microscopy. J Neurol Sci. (2016) 368:326–33. 10.1016/j.jns.2016.06.067 27538658

[169] Wang KC Tang SC Lee JE Tsai JC Lai DM Lin WC. Impaired microcirculation after subarachnoid hemorrhage in an *in vivo* animal model. Sci Rep. (2018) 8:1–15. 10.1038/s41598-018-31709-7 30190518 PMC6127197

[170] Balbi M Koide M Wellman GC Plesnila N. Inversion of neurovascular coupling after subarachnoid hemorrhage *in vivo*. J Cereb Blood Flow Metab. (2017) 37:3625–34. 10.1177/0271678X16686595 28112024 PMC5669344

[171] Graham DI Adams JH. Ischaemic brain damage in fatal head injuries. Lancet. (1971) 1:265–6. 10.1016/S0140-6736(71)91003-8 4100017

[172] Bragin DE Bush RC Nemoto EM. Effect of cerebral perfusion pressure on cerebral cortical microvascular shunting at high intracranial pressure in rats. Stroke. (2013) 44:177–81. 10.1161/STROKEAHA.112.668293 23204051 PMC3586667

[173] Martin NA Patwardhan R V Alexander MJ Africk CZ Lee JH Shalmon E. Characterization of cerebral hemodynamic phases following severe head trauma: hypoperfusion, hyperemia, and vasospasm. J Neurosurg. (1997) 87:9–19. 10.3171/jns.1997.87.1.0009 9202259

[174] Østergaard L Engedal TS Aamand R Mikkelsen R Iversen NK Anzabi M. Capillary transit time heterogeneity and flow-metabolism coupling after traumatic brain injury. J Cereb Blood Flow Metab. (2014) 34:1585–98. 10.1038/jcbfm.2014.131 25052556 PMC4269727

[175] Villaseñor R Kuennecke B Ozmen L Ammann M Kugler C Grüninger F. Region-specific permeability of the blood–brain barrier upon pericyte loss. J Cereb Blood Flow Metab. (2017) 37:3683–94. 10.1177/0271678X17697340 28273726 PMC5718326

[176] Schwarzmaier SM Gallozzi M Plesnila N. Identification of the vascular source of vasogenic brain edema following traumatic brain injury using *in vivo* 2-photon microscopy in mice. J Neurotr. (2015) 32:990–1000. 10.1089/neu.2014.3775 25585052

[177] Buckley EM Miller BF Golinski JM Sadeghian H McAllister LM Vangel M. Decreased microvascular cerebral blood flow assessed by diffuse correlation spectroscopy after repetitive concussions in mice. J Cereb Blood Flow Metab. (2015) 35:1995–2000. 10.1038/jcbfm.2015.161 26154866 PMC4671120

[178] Bellapart J Abi-Fares C Cuthbertson K Dunster K Diab S Platts DG. Cerebral microcirculation during mild head injury after a contusion and acceleration experimental model in sheep. Brain Inj. (2016) 30:1542–51. 10.1080/02699052.2016.1199894 27564238

[179] Hartings JA York J Carroll CP Hinzman JM Mahoney E Krueger B. Subarachnoid blood acutely induces spreading depolarizations and early cortical infarction. Brain. (2017) 140:2673–90. 10.1093/brain/awx214 28969382 PMC5841026

[180] Eriksen N Rostrup E Fabricius M Scheel M Major S Winkler MKL. Early focal brain injury after subarachnoid hemorrhage correlates with spreading depolarizations. Neurology. (2019) 92:e326–41. 10.1212/WNL.0000000000006814 30593517

[181] Strong AJ Anderson PJ Watts HR Virley DJ Lloyd A Irving EA. Peri-infarct depolarizations lead to loss of perfusion in ischaemic gyrencephalic cerebral cortex. Brain. (2006) 130:995–1008. 10.1093/brain/awl392 17438018

[182] Woitzik J Hecht N Pinczolits A Sandow N Major S Winkler MKL. Propagation of cortical spreading depolarization in the human cortex after malignant stroke. Neurology. (2013) 80:1095–102. 10.1212/WNL.0b013e3182886932 23446683

[183] Eikermann-Haerter K Hyun Lee J Yuzawa I Liu CH Zhou Z Kyoung Shin H. Migraine mutations increase stroke vulnerability by facilitating ischemic depolarizations. Circulation. (2012) 125:335–45. 10.1161/CIRCULATIONAHA.111.045096 22144569 PMC3276214

[184] Kumagai T Walberer M Nakamura H Endepols H Sué M Vollmar S. Distinct spatiotemporal patterns of spreading depolarizations during early infarct evolution: evidence from real-time imaging. J Cereb Blood Flow Metab. (2011) 31:580–92. 10.1038/jcbfm.2010.128 20700132 PMC3049513

[185] Ayata C Lauritzen M. Spreading depression, spreading depolarizations, and the cerebral vasculature. Physiol Rev. (2015) 95:953–93. 10.1152/physrev.00027.2014 26133935 PMC4491545

[186] Østergaard L Dreier JP Hadjikhani N Jespersen SN Dirnagl U Dalkara T. Neurovascular coupling during cortical spreading depolarization and -depression. Stroke. (2015) 46:1392–401. 10.1161/STROKEAHA.114.008077 25882051

[187] Khennouf L Gesslein B Brazhe A Octeau JC Kutuzov N Khakh BS. Active role of capillary pericytes during stimulation-induced activity and spreading depolarization. Brain. (2018) 141:2032–46. 10.1093/brain/awy143 30053174 PMC6022680

[188] Shahim P Tegner Y Gustafsson B Gren M Ärlig J Olsson M. Neurochemical aftermath of repetitive mild traumatic brain injury. JAMA Neurol. (2016) 73:1308–15. 10.1001/jamaneurol.2016.2038 27654934

[189] Aldag M Armstrong RC Bandak F Bellgowan PSF Bentley T Biggerstaff S. The biological basis of chronic traumatic encephalopathy following blast injury: a literature review. J Neurotr. (2017) 34:S-26-S-43. 10.1089/neu.2017.5218 28937953 PMC5695742

[190] McKee AC Cantu RC Nowinski CJ Hedley-Whyte ET Gavett BE Budson AE. Chronic traumatic encephalopathy in athletes: progressive tauopathy after repetitive head injury. J Neuropathol Exp Neurol. (2009) 68:709–35. 10.1097/NEN.0b013e3181a9d503 19535999 PMC2945234

[191] McKee AC Stein TD Kiernan PT Alvarez VE. The neuropathology of chronic traumatic encephalopathy. Brain Pathol. (2015) 25:350–64. 10.1111/bpa.12248 25904048 PMC4526170

[192] Sahyouni R Gutierrez P Gold E Robertson RT Cummings BJ. Effects of concussion on the blood-brain barrier in humans and rodents. J Concussion. (2017). 10.1177/2059700216684518. [Epub ahead of print]. 30828466 PMC6391889

[193] Tagge CA Fisher AM Minaeva OV Gaudreau-Balderrama A Moncaster JA Zhang XL. Concussion, microvascular injury, and early tauopathy in young athletes after impact head injury and an impact concussion mouse model. Brain. (2018) 141:422–58. 10.1093/brain/awx350 29360998 PMC5837414

[194] Goldstein LE Fisher AM Tagge CA Zhang XL Velisek L Sullivan JA. Chronic traumatic encephalopathy in blast-exposed military veterans and a blast neurotrauma mouse model. Sci Transl Med. (2012) 4:134ra60. 10.1016/j.jalz.2012.05.592 22593173 PMC3739428

[195] Zanier ER Bertani I Sammali E Pischiutta F Chiaravalloti MA Vegliante G. Induction of a transmissible tau pathology by traumatic brain injury. Brain. (2018) 141:2685–99. 10.1093/brain/awy193 30084913 PMC6113646

[196] Wolters FJ Zonneveld HI Hofman A van der Lugt A Koudstaal PJ Vernooij MW Heart-brain connection collaborative research group. cerebral perfusion and the risk of dementia. Circulation. (2017) 136:719–28. 10.1161/CIRCULATIONAHA.117.027448 28588075

[197] Dai W Lopez OL Carmichael OT Becker JT Kuller LH Gach HM. Mild cognitive impairment and alzheimer disease: patterns of altered cerebral blood flow at MR imaging. Radiology. (2009) 250:856–66. 10.1148/radiol.2503080751 19164119 PMC2680168

[198] Iturria-Medina Y Sotero RC Toussaint PJ Mateos-Pérez JM Evans AC Alzheimer's disease neuroimaging initiative. Early role of vascular dysregulation on late-onset Alzheimer's disease based on multifactorial data-driven analysis. Nat Commun. (2016) 7:11934. 10.1038/ncomms11934 27327500 PMC4919512

[199] Santos CY Snyder PJ Wu WC Zhang M Echeverria A Alber J. Pathophysiologic relationship between Alzheimer's disease, cerebrovascular disease, and cardiovascular risk: a review and synthesis. Alzheimer's Dement. (2017) 7:69–87. 10.1016/j.dadm.2017.01.005 28275702 PMC5328683

[200] Qiu L Ng G Tan EK Liao P Kandiah N Zeng L Chronic cerebral hypoperfusion enhances Tau hyperphosphorylation and reduces autophagy in Alzheimers disease mice. Sci Rep. (2016) 6:23964 10.1038/srep23964 27050297 PMC4822118

[201] Pluta R Bogucka-Kocka A Ułamek-Kozioł M Bogucki J Januszewski S Kocki J. Ischemic tau protein gene induction as an additional key factor driving development of Alzheimer's phenotype changes in CA1 area of hippocampus in an ischemic model of Alzheimer's disease. Pharmacol Rep. (2018) 70:881–84. 10.1016/j.pharep.2018.03.004 30096486

[202] Kuroiwa T. Experimental cerebral ischemia: the contribution of the Bethesda Group. Acta Neurochir Suppl. (2010) 106:17–9. 10.1007/978-3-211-98811-4_2 19812914

[203] Farkas E De Jong GI De Vos RAI Jansen Steur ENH Luiten PGM. Pathological features of cerebral cortical capillaries are doubled in Alzheimer's disease and Parkinson's disease. Acta Neuropathol. (2000) 100:395–402. 10.1007/s004010000195 10985698

[204] Nielsen RB Egefjord L Angleys H Mouridsen K Gejl M Møller A. Capillary dysfunction is associated with symptom severity and neurodegeneration in Alzheimer's disease. Alzheimer Dement. (2017) 13:1143–53. 10.1016/j.jalz.2017.02.007 28343848

[205] Gutiérrez-Jiménez E Angleys H Rasmussen PM West MJ Catalini L Iversen NK. Disturbances in the control of capillary flow in an aged APPswe/PS1ΔE9 model of Alzheimer's disease. Neurobiol Aging. (2018) 62:82–94. 10.1016/j.neurobiolaging.2017.10.006 29131981

[206] Reeson P Choi K Brown CE. VEGF signaling regulates the fate of obstructed capillaries in mouse cortex. Elife. (2018) 7:e33670. 10.7554/eLife.33670 29697373 PMC5919759

[207] Park L Uekawa K Garcia-Bonilla L Koizumi K Murphy M Pistik R. Brain perivascular macrophages initiate the neurovascular dysfunction of alzheimer Aβ peptides. Circ Res. (2017) 121:258–69. 10.1161/CIRCRESAHA.117.311054 28515043 PMC5522360

[208] Jaworski T Lechat B Demedts D Gielis L Devijver H Borghgraef P. Dendritic degeneration, neurovascular defects, and inflammation precede neuronal loss in a mouse model for tau-mediated neurodegeneration. Am J Pathol. (2011) 179:2001–15. 10.1016/j.ajpath.2011.06.025 21839061 PMC3181369

[209] Sagare AP Bell RD Zhao Z Ma Q Winkler EA Ramanathan A. Pericyte loss influences Alzheimer-like neurodegeneration in mice. Nat Commun. (2013) 4:1–14. 10.1038/ncomms3932 24336108 PMC3945879

[210] Sengillo JD Winkler EA Walker CT Sullivan JS Johnson M Zlokovic B V. Deficiency in mural vascular cells coincides with blood-brain barrier disruption in Alzheimer's disease. Brain Pathol. (2013) 23:303–10. 10.1111/bpa.12004 23126372 PMC3628957

[211] Montagne A Nikolakopoulou AM Zhao Z Sagare AP Si G Lazic D. Pericyte degeneration causes white matter dysfunction in the mouse central nervous system. Nat Med. (2018) 24:326–37. 10.1038/nm.4482 29400711 PMC5840035

[212] Wilhelmus MMM Otte-Höller I van Triel JJJ Veerhuis R Maat-Schieman MLC Bu G. Lipoprotein receptor-related protein-1 mediates amyloid-beta-mediated cell death of cerebrovascular cells. Am J Pathol. (2007) 171:1989–99. 10.2353/ajpath.2007.070050 18055545 PMC2111121

[213] Rensink AAM Verbeek MM Otte-Höller I ten Donkelaar HT de Waal RMW Kremer B. Inhibition of amyloid-beta-induced cell death in human brain pericytes *in vitro*. Brain Res. (2002) 952:111–21. 10.1016/S0006-8993(02)03218-3 12363410

[214] Verbeek MM Otte-Höller I Ruiter DJ de Waal RM. Human brain pericytes as a model system to study the pathogenesis of cerebrovascular amyloidosis in Alzheimer's disease. Cell Mol Biol. (1999) 45:37–46. 10099838

[215] Toth P Tucsek Z Sosnowska D Gautam T Mitschelen M Tarantini S. Age-related autoregulatory dysfunction and cerebromicrovascular injury in mice with angiotensin II-induced hypertension. J Cereb Blood Flow Metab. (2013) 33:1732–42. 10.1038/jcbfm.2013.143 23942363 PMC3824186

[216] Kruyer A Soplop N Strickland S Norris EH. Chronic hypertension leads to neurodegeneration in the TgSwDI mouse model of alzheimer's disease. Hypertens. (2015) 66:175–82. 10.1161/HYPERTENSIONAHA.115.05524 25941345 PMC4465852

[217] Thériault P ElAli A Rivest S. High fat diet exacerbates Alzheimer's disease-related pathology in APPswe/PS1 mice. Oncotarget. (2016) 7:67808–27. 10.18632/oncotarget.12179 27661129 PMC5356521

[218] Casey CS Atagi Y Yamazaki Y Shinohara M Tachibana M Fu Y. Apolipoprotein E inhibits cerebrovascular pericyte mobility through a RhoA protein-mediated pathway. J Biol Chem. (2015) 290:14208–17. 10.1074/jbc.M114.625251 25903128 PMC4447989

[219] Halliday MR Rege SV Ma Q Zhao Z Miller CA Winkler EA. Accelerated pericyte degeneration and blood-brain barrier breakdown in apolipoprotein E4 carriers with Alzheimer's disease. J Cereb Blood Flow Metab. (2016) 36:216–27. 10.1038/jcbfm.2015.44 25757756 PMC4758554

[220] Zlokovic BV. Cerebrovascular effects of apolipoprotein E: implications for Alzheimer disease. JAMA Neurol. (2013) 70:440–4. 10.1001/jamaneurol.2013.2152 23400708 PMC4414030

[221] Vidal R Calero M Piccardo P Farlow MR Unverzagt FW Méndez E Senile dementia associated with amyloid beta protein angiopathy and tau perivascular pathology but not neuritic plaques in patients homozygous for the APOE-epsilon4 allele. Acta Neuropathol. (2000) 100:1–12. 10.1007/s004010051186 10912914

[222] Kolinko Y Krakorova K Cendelin J Tonar Z Kralickova M. Microcirculation of the brain: morphological assessment in degenerative diseases and restoration processes. Rev Neurosci. (2014) 26:75–93. 10.1515/revneuro-2014-0049 25337818

[223] Guan J Pavlovic D Dalkie N Waldvogel HJ O'Carroll SJ Green CR Vascular degeneration in parkinsons disease. Brain Pathol. (2013) 23:154–64. 10.1111/j.1750-3639.2012.00628.x 22897695 PMC8029297

[224] Sarkar S Raymick J Mann D Bowyer J Hanig J Schmued L. Neurovascular changes in acute, sub-acute and chronic mouse models of parkinson's disease. Curr Neurovasc Res. (2014) 11:48–61. 10.2174/1567202610666131124234506 24274908

[225] Shibuya K Yagishita S Nakamura A Uchihara T. Perivascular orientation of astrocytic plaques and tuft-shaped astrocytes. Brain Res. (2011) 1404:50–4. 10.1016/j.brainres.2011.06.014 21722877

[226] Desai Bradaric B Patel A Schneider JA Carvey PM Hendey B. Evidence for angiogenesis in Parkinson's disease, incidental Lewy body disease, and progressive supranuclear palsy. J Neural Transm. (2012) 119:59–71. 10.1007/s00702-011-0684-8 21748523 PMC3352316

[227] Wong PC Pardo CA Borchelt DR Lee MK Copeland NG Jenkins NA. An adverse property of a familial ALS-linked SOD1 mutation causes motor neuron disease characterized by vacuolar degeneration of mitochondria. Neuron. (1995) 14:1105–16. 10.1016/0896-6273(95)90259-7 7605627

[228] Caccamo A Majumder S Oddo S. Cognitive decline typical of frontotemporal lobar degeneration in transgenic mice expressing the 25-kDa C-terminal fragment of TDP-43. Am J Pathol. (2012) 180:293–302. 10.1016/j.ajpath.2011.09.022 22067910 PMC3338346

[229] Qiu H Lee S Shang Y Wang WY Au KF Kamiya S. ALS-associated mutation FUS-R521C causes DNA damage and RNA splicing defects. J Clin Invest. (2014) 124:981–99. 10.1172/JCI72723 24509083 PMC3938263

[230] O'Rourke JG Bogdanik L Muhammad AKMG Gendron TF Kim KJ Austin A. C9orf72 BAC transgenic mice display typical pathologic features of ALS/FTD. Neuron. (2015) 88:892–901. 10.1016/j.neuron.2015.10.027 26637796 PMC4672384

[231] Miyazaki K Ohta Y Nagai M Morimoto N Kurata T Takehisa Y. Disruption of neurovascular unit prior to motor neuron degeneration in amyotrophic lateral sclerosis. J Neurosci Res. (2011) 89:718–28. 10.1002/jnr.22594 21337372

[232] Miyazaki K Masamoto K Morimoto N Kurata T Mimoto T Obata T. Early and progressive impairment of spinal blood flow-glucose metabolism coupling in motor neuron degeneration of ALS model mice. J Cereb Blood Flow Metab. (2012) 32:456–67. 10.1038/jcbfm.2011.155 22068226 PMC3293114

[233] Abrahams S Goldstein LH Kew JJ Brooks DJ Lloyd CM Frith CD. Frontal lobe dysfunction in amyotrophic lateral sclerosis. A PET study. Brain. (1996) 119(Pt 6):2105–20. 10.1093/brain/119.6.2105 9010014

[234] Ishikawa T Morita M Nakano I. Constant blood flow reduction in premotor frontal lobe regions in ALS with dementia – a SPECT study with 3D-SSP. Acta Neurol Scand. (2007) 116:340–4. 10.1111/j.1600-0404.2007.00876.x 17922728

[235] van Es MA Hardiman O Chio A Al-Chalabi A Pasterkamp RJ Veldink JH. Amyotrophic lateral sclerosis. Lancet. (2017) 390:2084–98. 10.1016/S0140-6736(17)31287-4 28552366

[236] Winkler EA Sengillo JD Sullivan JS Henkel JS Appel SH Zlokovic BV. Blood-spinal cord barrier breakdown and pericyte reductions in amyotrophic lateral sclerosis. Acta Neuropathol. (2013) 125:111–20. 10.1007/s00401-012-1039-8 22941226 PMC3535352

[237] Zhong Z Deane R Ali Z Parisi M Shapovalov Y O'Banion MK. ALS-causing SOD1 mutants generate vascular changes prior to motor neuron degeneration. Nat Neurosci. (2008) 11:420–2. 10.1038/nn2073 18344992 PMC2895310

[238] Meldrum BS. Concept of activity-induced cell death in epilepsy: historical and contemporary perspectives. Prog Brain Res. (2002) 135:3–11. 10.1016/S0079-6123(02)35003-9 12143350

[239] Farrell JS Wolff MD Teskey GC. Neurodegeneration and Pathology in Epilepsy: Clinical and Basic Perspectives. In: Advances in Neurobiology. (2017). p. 317–34. 10.1007/978-3-319-57193-5_12 28674987

[240] Mehta A Prabhakar M Kumar P Deshmukh R Sharma PL. Excitotoxicity: bridge to various triggers in neurodegenerative disorders. Eur J Pharmacol. (2013) 698:6–18. 10.1016/j.ejphar.2012.10.032 23123057

[241] Ko SB Ortega-Gutierrez S Choi HA Claassen J Presciutti M Schmidt JM. Status epilepticus–induced hyperemia and brain tissue hypoxia after cardiac arrest. Arch Neurol. (2011) 68:1323–6. 10.1001/archneurol.2011.240 21987548

[242] Johnson AC Cipolla MJ. Altered hippocampal arteriole structure and function in a rat model of preeclampsia: potential role in impaired seizure-induced hyperemia. J Cereb Blood Flow Metab. (2017) 37:2857–69. 10.1177/0271678X16676287 27815419 PMC5536792

[243] Wichert-Ana L de Azevedo-Marques PM Oliveira LF Terra-Bustamante VC Fernandes RMF Santos AC. Interictal hyperemia correlates with epileptogenicity in polymicrogyric cortex. Epilepsy Res. (2008) 79:39–48. 10.1016/j.eplepsyres.2007.12.018 18291625

[244] Leal-Campanario R Alarcon-Martinez L Rieiro H Martinez-Conde S Alarcon-Martinez T Zhao X. Abnormal capillary vasodynamics contribute to ictal neurodegeneration in epilepsy. Sci Rep. (2017) 7:43276. 10.1038/srep43276 28240297 PMC5327474

[245] Taskiran-Sag A Yemisci M Gursoy-Ozdemir Y Erdener SE Karatas H Yuce D. Improving microcirculatory reperfusion reduces parenchymal oxygen radical formation and provides neuroprotection. Stroke. (2018) 49:1267–75. 10.1161/STROKEAHA.118.020711 29669868

[246] Jung KH Chu K Ko SY Lee ST Sinn DI Park DK. Early intravenous infusion of sodium nitrite protects brain against *in vivo* ischemia-reperfusion injury. Stroke. (2006) 37:2744–50. 10.1161/01.STR.0000245116.40163.1c 17008610

[247] Fathi AR Pluta RM Bakhtian KD Qi M Lonser RR. Reversal of cerebral vasospasm via intravenous sodium nitrite after subarachnoid hemorrhage in primates. J Neurosurg. (2011) 115:1213–20. 10.3171/2011.7.JNS11390 21888479 PMC4749030

[248] Cantow K Flemming B Ladwig-Wiegard M Persson PB Seeliger E. Low dose nitrite improves reoxygenation following renal ischemia in rats. Sci Rep. (2017) 7:14597. 10.1038/s41598-017-15058-5 29097777 PMC5668317

[249] Terpolilli NA Kim SW Thal SC Kataoka H Zeisig V Nitzsche B. Inhalation of nitric oxide prevents ischemic brain damage in experimental stroke by selective dilatation of collateral arterioles. Circ Res. (2012) 110:727–38. 10.1161/CIRCRESAHA.111.253419 22207711

[250] Terpolilli NA Kim SW Thal SC Kuebler WM Plesnila N. Inhaled nitric oxide reduces secondary brain damage after traumatic brain injury in mice. J Cereb Blood Flow Metab. (2013) 33:311–8. 10.1038/jcbfm.2012.176 23188422 PMC3564204

[251] Garry PS Ezra M Rowland MJ Westbrook J Pattinson KTS. The role of the nitric oxide pathway in brain injury and its treatment–from bench to bedside. Exp Neurol. (2015) 263:235–43. 10.1016/j.expneurol.2014.10.017 25447937

[252] Terpolilli NA Feiler S Dienel A Müller F Heumos N Friedrich B. Nitric oxide inhalation reduces brain damage, prevents mortality, and improves neurological outcome after subarachnoid hemorrhage by resolving early pial microvasospasms. J Cereb Blood Flow Metab. (2016) 36:2096–107. 10.1177/0271678X15605848 26661144 PMC5363657

[253] Li J Iadecola C. Nitric oxide and adenosine mediate vasodilation during functional activation in cerebellar cortex. Neuropharmacology. (1994) 33:1453–61. 10.1016/0028-3908(94)90049-3 7532829

[254] O'Farrell FM Mastitskaya S Hammond-Haley M Freitas F Wah WR Attwell D. Capillary pericytes mediate coronary no-reflow after myocardial ischaemia. Elife. (2017) 6:e29280. 10.7554/eLife.29280 29120327 PMC5705208

[255] Costa MA Paiva AE Andreotti JP Cardoso MV Cardoso CD Mintz A. Pericytes constrict blood vessels after myocardial ischemia. J Mol Cell Cardiol. (2018) 116:1–4. 10.1016/j.yjmcc.2018.01.014 29371134 PMC6089363

[256] Dalkara T Alarcon-Martinez L. Cerebral microvascular pericytes and neurogliovascular signaling in health and disease. Brain Res. (2015) 1623:3–17. 10.1016/j.brainres.2015.03.047 25862573

[257] Biaggioni I Olafsson B Robertson RM Hollister AS Robertson D. Cardiovascular and respiratory effects of adenosine in conscious man. Evidence for chemoreceptor activation. Circ Res. (1987) 61:779–86. 10.1161/01.RES.61.6.779 3677336

[258] Abreu A Mahmarian JJ Nishimura S Boyce TM Verani MS. Tolerance and safety of pharmacologic coronary vasodilation with adenosine in association with thallium-201 scintigraphy in patients with suspected coronary artery disease. J Am Coll Cardiol. (1991) 18:730–5. 10.1016/0735-1097(91)90796-C 1869736

[259] Gaston B. Summary: systemic effects of inhaled nitric oxide. Proc Am Thorac Soc. (2006) 3:170–2. 10.1513/pats.200506-049BG 16565427

[260] den Uil CA Brugts JJ. Impact of intravenous nitroglycerin in the management of acute decompensated heart failure. Curr Heart Fail Rep. (2015) 12:87–93. 10.1007/s11897-014-0230-8 25301529

[261] Seabra AB Duran N. Nanoparticulated nitric oxide donors and their biomedical applications. Mini Rev Med Chem. (2017) 17:216–23. 10.2174/1389557516666160808124624 27515711

[262] Cheng J Korte N Nortley R Sethi H Tang Y Attwell D. Targeting pericytes for therapeutic approaches to neurological disorders. Acta Neuropathol. (2018) 136:507–23. 10.1007/s00401-018-1893-0 30097696 PMC6132947

[263] Sugiyama T Kawamura H Yamanishi S Kobayashi M Katsumura K Puro DG Regulation of P2X _7_ -induced pore formation and cell death in pericyte-containing retinal microvessels. Am J Physiol. (2005) 288:C568–76. 10.1152/ajpcell.00380.2004 15496477

[264] Wu DM Kawamura H Sakagami K Kobayashi M Puro DG. Cholinergic regulation of pericyte-containing retinal microvessels. Am J Physiol Circ Physiol. (2003) 284:H2083–90. 10.1152/ajpheart.01007.2002 12560212

[265] Kutcher ME Herman IM. The pericyte: cellular regulator of microvascular blood flow. Microvasc Res. (2009) 77:235–46. 10.1016/j.mvr.2009.01.007 19323975 PMC2668721

[266] Niego B Lee N Larsson P De Silva TM Au AE-L McCutcheon F. Selective inhibition of brain endothelial Rho-kinase-2 provides optimal protection of an *in vitro* blood-brain barrier from tissue-type plasminogen activator and plasmin. PLoS ONE. (2017) 12:e0177332. 10.1371/journal.pone.0177332 28510599 PMC5433693

[267] Shin HK Salomone S Potts EM Lee SW Millican E Noma K. Rho-kinase inhibition acutely augments blood flow in focal cerebral ischemia via endothelial mechanisms. J Cereb Blood Flow Metab. (2007) 27:998–1009. 10.1038/sj.jcbfm.9600406 17033691 PMC2614438

[268] Hyun Lee J Zheng Y von Bornstadt D Wei Y Balcioglu A Daneshmand A Selective ROCK2 inhibition in focal cerebral ischemia. Ann Clin Transl Neurol. (2014) 1:2–14. 10.1002/acn3.19 24466563 PMC3900310

[269] Shin HK Huang PL Ayata C. Rho-Kinase inhibition improves ischemic perfusion deficit in hyperlipidemic mice. J Cereb Blood Flow Metab. (2014) 34:284–87. 10.1038/jcbfm.2013.195 24192634 PMC3915205

[270] Iba T Levy JH Hirota T Hiki M Sato K Murakami T. Protection of the endothelial glycocalyx by antithrombin in an endotoxin-induced rat model of sepsis. Thromb Res. (2018) 171:1–6. 10.1016/j.thromres.2018.09.042 30216821

[271] Iba T Levy JH. Derangement of the endothelial glycocalyx in sepsis. J Thromb Haemost. (2019) 17:283–94. 10.1111/jth.14371 30582882

[272] Gresele P Momi S Falcinelli E. Anti-platelet therapy: phosphodiesterase inhibitors. Br J Clin Pharmacol. (2011) 72:634–46. 10.1111/j.1365-2125.2011.04034.x 21649691 PMC3195739

[273] Muravyov AV Yakusevich VV Chuchkanov FA Maimistova AA Bulaeva SV Zaitsev LG. Hemorheological efficiency of drugs, targeting on intracellular phosphodiesterase activity: *in vitro* study. Clin Hemorheol Microcirc. (2007) 36:327–34. 17502703

[274] Zhang Q Wang G Yuan W Wu J Wang M Li C. The effects of phosphodiesterase-5 inhibitor sildenafil against post-resuscitation myocardial and intestinal microcirculatory dysfunction by attenuating apoptosis and regulating microRNAs expression: essential role of nitric oxide syntheses signaling. J Transl Med. (2015) 13:177. 10.1186/s12967-015-0550-9 26040988 PMC4467614

[275] Magny E Le Petitcorps H Pociumban M Bouksani-Kacher Z Pautas É Belmin J. Predisposing and precipitating factors for delirium in community-dwelling older adults admitted to hospital with this condition: a prospective case series. PLoS ONE. (2018) 13:e0193034. 10.1371/journal.pone.0193034 29474380 PMC5825033

[276] Jickling GC Liu D Ander BP Stamova B Zhan X Sharp FR. Targeting neutrophils in ischemic stroke: translational insights from experimental studies. J Cereb Blood Flow Metab. (2015) 35:888–901. 10.1038/jcbfm.2015.45 25806703 PMC4640255

[277] Provencio JJ Altay T Smithason S Moore SK Ransohoff RM. Depletion of Ly6G/C(+) cells ameliorates delayed cerebral vasospasm in subarachnoid hemorrhage. J Neuroimmunol. (2011) 232:94–100. 10.1016/j.jneuroim.2010.10.016 21059474 PMC3053416

[278] Herz J Sabellek P Lane TE Gunzer M Hermann DM Doeppner TR. Role of neutrophils in exacerbation of brain injury after focal cerebral ischemia in hyperlipidemic mice. Stroke. (2015) 46:2916–25. 10.1161/STROKEAHA.115.010620 26337969 PMC4589522

[279] Lee PY Wang JX Parisini E Dascher CC Nigrovic PA. Ly6 family proteins in neutrophil biology. J Leukoc Biol. (2013) 94:585–94. 10.1189/jlb.0113014 23543767

[280] Hu F Wang Y Gong K Ge G Cao M Zhao P. Protective effects of drag-reducing polymers on ischemic reperfusion injury of isolated rat heart. Clin Hemorheol Microcirc. (2016) 62:1–11. 10.3233/CH-151925 25633566

[281] Bragin DE Peng Z Bragina OA Statom GL Kameneva MV Nemoto EM. Improvement of impaired cerebral microcirculation using rheological modulation by drag-reducing polymers. Adv Exp Med Biol. (2016) 923:239–44. 10.1007/978-3-319-38810-6_32 27526149 PMC4988339

[282] Bragin DE Kameneva MV Bragina OA Thomson S Statom GL Lara DA. Rheological effects of drag-reducing polymers improve cerebral blood flow and oxygenation after traumatic brain injury in rats. J Cereb Blood Flow Metab. (2017) 37:762–75. 10.1177/0271678X16684153 28155574 PMC5363490

[283] Subramani K Raju SP Chu X Warren M Pandya CD Hoda N. Effect of plasma-derived extracellular vesicles on erythrocyte deformability in polymicrobial sepsis. Int Immunopharmacol. (2018) 65:244–47. 10.1016/j.intimp.2018.10.011 30340103

[284] Sorkin R Bergamaschi G Kamsma D Brand G Dekel E Ofir-Birin Y. Probing cellular mechanics with acoustic force spectroscopy. Mol Biol Cell. (2018) 29:2005–11. 10.1091/mbc.E18-03-0154 29927358 PMC6232971

